# Ultra-thin layered double hydroxide-mediated photothermal therapy combine with asynchronous blockade of PD-L1 and NR2F6 inhibit hepatocellular carcinoma

**DOI:** 10.1186/s12951-022-01565-9

**Published:** 2022-07-30

**Authors:** Yuan-Fei Lu, Jia-Ping Zhou, Qiao-Mei Zhou, Xiao-Yan Yang, Xiao-Jie Wang, Jie-Ni Yu, Jin-Guo Zhang, Yong-Zhong Du, Ri-Sheng Yu

**Affiliations:** 1 Department of Radiology, Second Affiliated Hospital, School of Medicine, Zhejiang University, 88 Jiefang Road, Hangzhou, 310009 Zhejiang People’s Republic of China; 2 Institute of Pharmaceutics, College of Pharmaceutical Sciences, Zhejiang University, 866 Yuhangtang Road, Hangzhou, 310058 Zhejiang People’s Republic of China

**Keywords:** Hepatocellular carcinoma, NR2F6, PD-L1 blockade, Photothermal therapy

## Abstract

**Background:**

The efficacy of immune checkpoint blockade (ICB), in the treatment of hepatocellular carcinoma (HCC), is limited due to low levels of tumor-infiltrating T lymphocytes and deficient checkpoint blockade in this immunologically "cool" tumor. Thus, combination approaches are needed to increase the response rates of ICB and induce synergistic antitumor immunity.

**Methods:**

Herein, we designed a pH-sensitive multifunctional nanoplatform based on layered double hydroxides (LDHs) loaded with siRNA to block the intracellular immune checkpoint NR2F6, together with the asynchronous blockade surface receptor PD-L1 to induce strong synergistic antitumor immunity. Moreover, photothermal therapy (PTT) generated by LDHs after laser irradiation modified an immunologically “cold” microenvironment to potentiate *Nr2f6*-siRNA and anti-PD-L1 immunotherapy. Flow cytometry was performed to assess the immune responses initiated by the multifunctional nanoplatform.

**Results:**

Under the slightly acidic tumor extracellular environment, PEG detached and the re-exposed positively charged LDHs enhanced tumor accumulation and cell uptake. The accumulated siRNA suppressed the signal of dual protumor activity in both immune and H22 tumor cells by silencing the NR2F6 gene, which further reduced the tumor burden and enhanced systemic antitumor immunity. The responses include enhanced tumor infiltration by CD4^+^ helper T cells, CD8^+^ cytotoxic T cells, and mature dendritic cells; the significantly decreased level of immune suppressed regulator T cells. The therapeutic responses were also attributed to the production of IL-2, IFN-γ, and TNF-α. The prepared nanoparticles also exhibited potential magnetic resonance imaging (MRI) ability, which could serve to guide synergistic immunotherapy treatment.

**Conclusions:**

In summary, the three combinations of PTT, NR2F6 gene ablation and anti-PD-L1 can promote a synergistic immune response to inhibit the progression of primary HCC tumors and prevent metastasis. This study can be considered a proof-of-concept for the targeting of surface and intracellular immune checkpoints to supplement the existing HCC immunotherapy treatments.

**Supplementary Information:**

The online version contains supplementary material available at 10.1186/s12951-022-01565-9.

## Background

Currently, hepatocellular carcinoma (HCC) is one of the most prevalent cancers and a leading cause of cancer death globally [1]. The majority of patients with HCC proceed to advanced stages when diagnosed, and the high risk of recurrence and metastasis (up to 70% at 5 years) is the dominant challenge in the clinical management of HCC [2, 3]. Patients diagnosed at early stages are suitable candidates for radical resection combined with systemic therapy, whereas patients at more advanced stages are not eligible for surgical treatment, and chemoembolization has shown limited benefits [2]. Cancer immunotherapy has recently emerged as a viable treatment option for both primary and metastatic cancers [4–6]. Immune checkpoint blockade (ICB), which blocks immunosuppressive ligand–receptor interactions (such as PD-1/PD-L1), is one of the most promising immunotherapeutic approaches [7]. Unfortunately, due to constant exposure to various antigens from the gastrointestinal system and blood, the liver is considered an immunosuppressive organ, which limits the applications of ICB [8–10]. In a large portion of HCC patients, the response rates generated by ICB are still very low [9]. Hence, combining ICB with other therapeutic treatments that can reverse “cold” HCC tumors into “hot” sites and remodel the immunosuppressive tumor microenvironment (TME) may increase the response rates of ICB and broaden the application of immunotherapy in primary and metastatic tumors.

Photothermal therapy (PTT) is a new cancer therapy paradigm that is noninvasive, precise, and controllable [11, 12]. Recently, mild PTT with a fever temperature (~ 42–45 °C) applied for tumor treatment was found to be effective [13]. Instead of killing tumor cells directly, mild PTT can overcome immunologically “cold” tumors and induce an immune-favorable TME that boosts innate immune responses [13]. Previous investigations have demonstrated that PTT can upregulate immunogenic cell death (ICD) biomarkers, promote the maturation of dendritic cells (DCs) and recruit tumor-infiltrating T lymphocytes (TILs) [14, 15]. However, some self-protection proteins, such as PD-L1, are upregulated by mild warmth. A growing number of studies have shown that combining PTT with ICB has therapeutic potential in cancer treatment [16–18].

Nuclear receptor subfamily 2, group F, member 6 (NR2F6) acts as an intracellular immune checkpoint in T cells and plays a negative regulatory role in T cell activation in cancer [19–21]. NR2F6 directly antagonizes NFAT/AP-1 complex DNA-binding capabilities on key cytokine promoters, such as *Il2*, *Ifng*, and *Tnfa* [20]. Thus, the ablation of genetic NR2F6 strongly enhances the secretion of interleukin-2 (IL-2), interferon-γ (IFN-γ), and tumor necrosis factor-α (TNF-α) both at tumor sites and ex vivo, thereby promoting antitumor immunological responses. In addition, NR2F6 is overexpressed in HCC tissues and promotes HCC development and progression [22]. Thus, *Nr2f6*-siRNA facilitates the apoptotic of HCC cells directly. Based on the dual protumor activity of NR2F6 in both immune and tumor cells, inhibition of NR2F6 may exert a unique and beneficial effect on HCC, realizing two birds with one stone. Furthermore, NR2F6 downregulation sensitizes tumors to established PD-1/PD-L1 axis blockade to prevent tumor progression due to the positive correlation between NR2F6 expression and the T-cell dysfunction/exhaustion phenotype [23]. Taken together, these data allowed us to hypothesize that the combination of genetic NR2F6 ablation and anti-PD-L1 (aPD-L1) immunotherapy with antitumor immune responses elicited by PTT may exert a strong synergistic effect, and thus present an effective strategy for HCC treatment.

Layered double hydroxide nanoparticles (LDHs) are composed of divalent and trivalent metal cations in the hydroxide layers with exchangeable anions intercalating between them [24, 25]. With the advantages of good biocompatibility, low cytotoxicity, tunable particle size, pH-controlled release, and protection of the genes in the interlayer [26–28], LDHs perform as an efficient siRNA delivery system in both T lymphocytes and tumor cells [29–32]. As reported previously, LDHs with different morphologies target different subcellular compartments [33]. Hexagonal LDHs have a proclivity for delivering siRNA to the perinuclear cytoplasm, where mRNA is degraded by siRNA. Furthermore, doping functional metal cations into LDHs, such as Cu(II) and Fe(III), allows strong photothermal conversion efficiency and magnetic resonance imaging (MRI) capability [24, 34]. However, LDHs easily aggregate in the physiological environment, which limits their applicability in vivo [35]. Thus, polyethylene glycol (PEG) coating is a promising strategy for maintaining the colloidal stability and dispersity of LDHs [36].

**Fig. 1:**
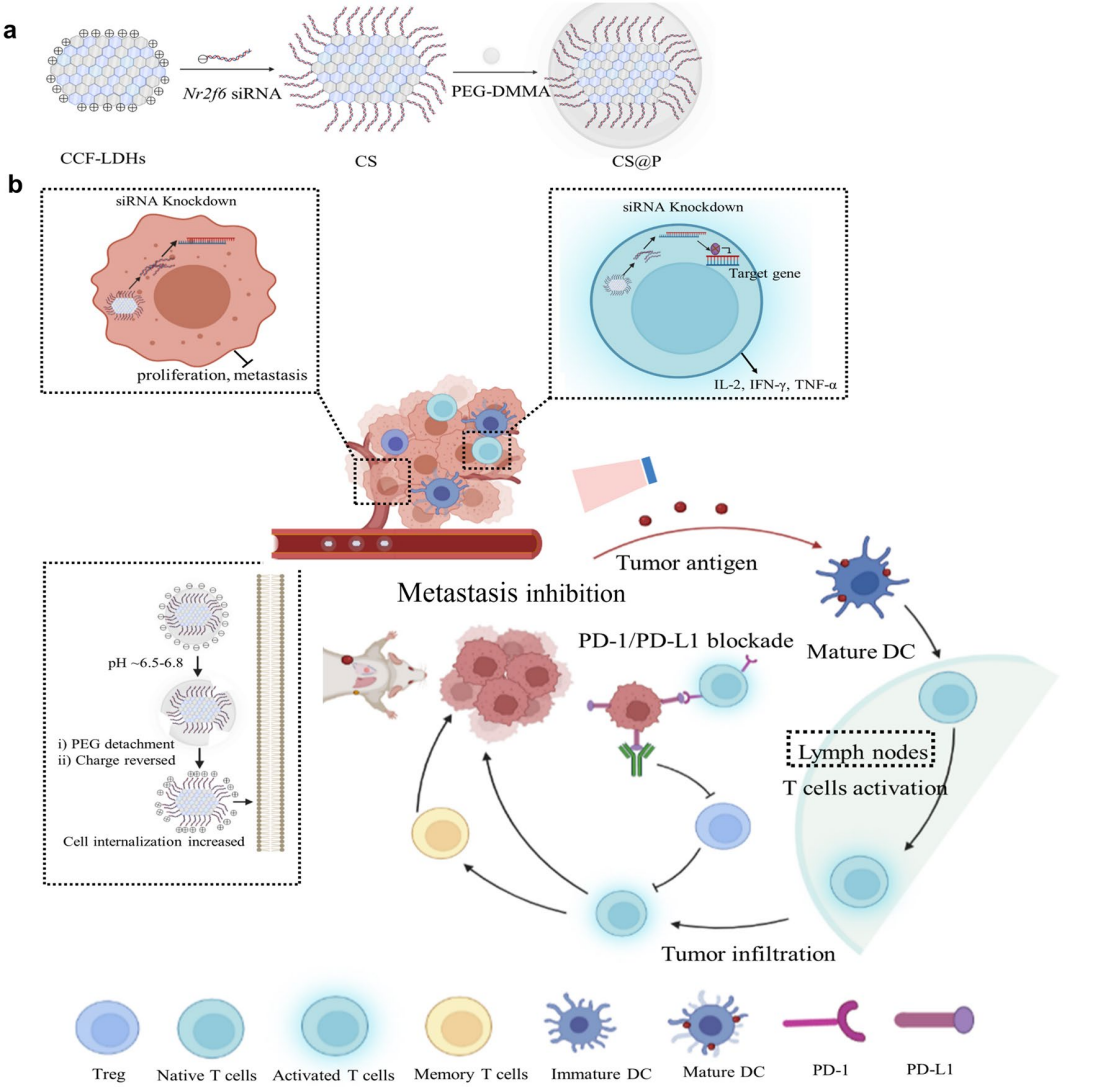
Schematic diagram of synthesis (**a**) and application in cancer treatment (**b**) of CS@P nanoparticles. **a** Preparation of pH-sensitive Nanoparticles CS@P used to deliver *Nr2f6* siRNA to both T cells and tumor cells. **b** Under NIR laser irradiation, fever temperature (~ 42–45 °C) reveres immunosuppressive TME and activates T cells. Via *Nr2f6* siRNA-mediated gene silencing, the pH- triggered nanoparticles CS@P not only suppress HCC cell proliferation and metastasis but also strongly enhance cytokines secretion of activated T cells, such as IL-2, IFN-γ, and TNF-α. The loss of NR2F6 and mild PTT further increase the response rates of established PD-1/PD-L1 checkpoint blockade to suppress primary and distant tumors, and prevent tumor metastasis

In this study, we designed pH-triggered nanoparticles based on Cu-doped LDHs (Cu-LDHs) to mitigate HCC progression by strengthening the weapon (T cells) and neutralizing the self-protection of tumor cells (Fig. 1). To this end, we developed PEG-dimethylmaleic anhydride (DMMA)-coated Cu-LDHs for *Nr2f6*-siRNA delivery (CS@P). Under the slightly acidic tumor extracellular environment (pH ~ 6.5–6.8), PEG-DMMA detached from the positively charged LDHs, and the re-exposed LDHs enhanced tumor accumulation and cell uptake [37, 38]. Upon laser irradiation, CS@P-mediated PTT induces ICD and recruits TILs, triggering the downstream immune responses. The accumulated siRNA blocks intracellular immune checkpoint NR2F6 to promote an immunogenic TME that allows powerful anti-tumor T-cell responses. We systematically tested the therapeutic effects of our multifunctional nanotheranostic CS@P in a H22 tumor model. The constructed CS@P may be able to activate antitumor immunity while also sensitizing HCC to PD-L1 blocking therapy. When used in conjunction with aPD-L1 medication, CS@P has the potential to not only inhibit the progression of primary tumors but also boost antitumor immune responses to suppress distant cancers and prevent tumor metastasis.

## Methods

### Materials

Cobalt nitrate hexahydrate (Co (NO3)2·6H2O, > 99.0%), cupric nitrate trihydrate (Cu(NO_3_)_2_·3H_2_O, > 99.0%), iron nitrate nonahydrate (Fe(NO3)3·9H2O, > 99.0%), sodium hydroxide (NaOH, > 98.0%), sodium nitrate (NaNO3, > 99.0%), methylene blue (MB), glutathione(GSH) and formamide were obtained from Aladdin Chemical. Co. Ltd (Shanghai, China). 2,3-dimethylmaleic anhydride (DMMA), mPEG_5k_-NH_2_, fluorescein isothiocyanate isomer (FITC) were obtained from Macklin Chemical. Co. Ltd (Shanghai, China). 2′, 7′-dichlorofluorescein diacetate (DCFH-DA), calcein acetoxymethyl ester (Calcein-AM) and propidium iodide (PI), Annexin V-FITC & PI apoptosis detection kit were acquired from Sigma-Aldrich Company (St. Louis, MO, USA). Phosphate buffer saline (PBS, 10 × , pH = 6.5;7.4), fetal bovine serum (FBS), 1640 medium were purchased from Procell Life Science&Technology Co.,Ltd (Wuhan, China). *Nr2fr6* siRNA, select negative siRNA were purchased from ThermoFisher (Waltham, MA, USA). PD-L1 antibody for i.v. injection was obtained from BioXCell (Beijing, China). PD-L1 and NR2F6 antibodies for flow cytometry and Western blot were obtained from Abcam (Cambridge, MA, USA). Flow cytometry antibodies for CD45, CD3, CD86, CD4, CD8, Foxp3, CD80 and CD11b were obtained from Biolegend (California, USA). IL-2, TNF-α, and IFN-γ ELISA kits were obtained from Mlbio (Shanghai, China). Milli-Q water was used throughout the experiments. Six-week-old female BALB/c mice were obtained from the Shanghai Silaike Laboratory Animal Co., Ltd.

### Preparation of CoCuFe-LDHs monolayer nanosheets

Firstly, Co(NO_3_)_2_·6H2O (3.2 mmol), Cu(NO_3_)_2_·3H_2_O (0.8 mmol) and Fe(NO_3_)_3_·9H2O (2 mmol) were dissolved in deionized water (40 mL) as solution A. Secondly, NaOH (2 mmol) was dissolved in another 30 mL deionized water to make solution B. Afterward, 10 mL of formamide was mixed with 30 mL deionized water as solution C. In the condition of 80 °C, solution A and solution B were slowly added into solution C within 30 min, under pH ~ 10. The resulting precipitation was centrifuged and washed four times with a mixture of deionized water and ethanol. The final CoCuFe-LDHs (CCF-LDHs) monolayer nanosheets were obtained after the removal of residual formamide through dialysis (8 kDa) for 48 h.

### Fabrication of CCF/PEG-DMMA

PEG_5K_-NH2 was dissolved in PBS (20 mL, ~ pH 8.5). DMMA was added to the solution slowly and reacted for 24 h at room temperature. The PEG-DMMA monolayer nanosheets were obtained through dialysis (8 kDa) for 48 h. CCF-LDHs (1 mg/mL) was added to prepared PEG-DMMA solution (1 mg/mL) under stirring for 4 h. The resulting mixture CCF/PEG-DMMA (C@P) was centrifuged and washed four times and resuspended in water for further use. CCF-siRNA/PEG-DMMA (CS@P) were prepared by a similar method as described above. In detail, CCF-siRNA (CCF-LDHs: 1 mg/mL; siRNA: 100 nM) was added to prepared PEG-DMMA solution (1 mg/mL) under stirring for 4 h, but using DEPC water in the whole process.

### Association of nucleic acid with CCF-LDHs

The siRNA loading capacity in CCF-LDHs was measured by mixing different w/w ratios of CCF-LDHs to siRNA (CS). The mixture was slightly agitated for 30 min at 37 °C. Prepare 2% agarose gel (containing Gel Red), followed by electrophoresed at 100 V for 30 min.

### Characterization

The hydrodynamic particle diameter distributions and potential of samples were obtained by a dynamic light scattering (DLS) method (LITESIZER 500, Anton-Paar, Austria) in triplicate. The morphologies of nanoparticles were observed in a transmission electronic microscopy (TEM; JEM-1200EX, JEOL, Japan). The Fourier transform infrared (FT-IR) spectra of nanoparticles were tested by a VECTOR 22 (Bruker, Germany) in the range of 400 to 4000 cm^−1^. X-ray diffraction patterns (D/MAX-2550 PC, Rigaku Inc., Japan) were recorded using Cu Kα radiation with the scan range from 10° to 80°. The chemical states of samples were analyzed by the X-ray photoelectron spectroscopy (XPS, EscaLab 250Xi, Thermo Fisher Scientific, USA). A field-emission scanning electron microscope–energy dispersive spectroscopy (FESEM–EDS, FEG650, FEI, USA) was applied to recorded element mapping images.

### Cell culture

H22 cells were incubated in RPMI 1640 containing 10% FBS and 1% penicillin–streptomycin at 37 °C.

### Cytotoxicity

H22 cells were cultured in a 96-well plate overnight. Afterwards, C@P or CCF-siRNA/PEG-DMMA (CS@P) with serious of concentrations were added and incubated with different pH (6.5 or 7.4) for another 24 h. Using Cell Counting Kit-8 (CCK-8) assay to determine cell viability. Otherwise, cells were suspended in 96-well plates overnight, followed by incubation under different conditions (C@P: 0–200 μg/mL, siRNA 0–200 nM, pH = 7.4 or 6.5, laser or not) for 24 h. Cytotoxicity assessment was same as above.

### Cell uptake of CCF-siRNA/PEG-DMMA

To evaluate the effects of cell internalization of CS@P, confocal laser scanning microscopy (CLSM) analysis was performed. H22 cells were cultured in a 6-well plate overnight at a density of 1 × 10^6^ cells per well. Then, the FITC-labeled nanoparticles were added into per well at different time points (1 h,4 h, 4 h + Laser) at pH 6.5 or 7.4. Afterward, the cells were washed three times with PBS solution and fixed in 4% paraformaldehyde for 15 min, followed by staining with DAPI for 15 min. Nikon microscope (Nikon Eclipse Ti-S, USA) was used to acquire images.

### Gene silencing in vitro

H22 and T cells were cultured in a 6-well plate overnight at a density of 1 × 10^6^ cells per well. CS was added to each well, and the cells were then transfected for another 24 h or 48 h at 37°. The targeted protein (NR2F6, PD-1, and PD-L1) was tested by Western blot analysis.

### Transwell assays

Cell migration and invasion assays were tested by 24-well chambers with 8 μm pore size. 5 × 10^4^ H22 cells in 100 μL of serum-free media were seeded in the upper chamber (pre-coated with Matrigel (BD) for invasion). 500 μL complete medium was added in the lower chamber. Cells on the top side of upper chambers were wiped and then the upper chambers were fixed and stained with 0.2% crystal violet. Cell numbers were counted using a microscope. This assay was repeated three times.

### In vitro and in vivo MR imaging

CCF-LDHs with series of concentration were dispersed for MRI measurements. In vivo, signal was obtained by injecting the nanoparticles via tail vein into mice at different time points.

### In vivo biodistribution

Tumor-bearing mice were divided into two groups and intravenously injected with CS@P or CS at different time points (0, 6, 12, 24, 48) post-injection, IVIS Spectrum in Vivo Imaging System (caliper, perkinelmer, USA) was used to monitor biodistribution. At the last time point, major organs (heart, liver, spleen, lung, kidneys and tumors) were collected for vitro biodistribution.

### In vitro and in vivo photothermal effect

To investigate the PTT ability of C@P, the solutions were exposed to the 808 nm laser (LASEVER INC., China). Pure water was irradiated as a control. The cuvettes containing different concentrations of C@P, were irradiated by 808 nm NIR laser at power of 1.0 w cm^−2^ for 5 min. The temperature variation of each sample was monitor by a thermocouple probe, and photographed by an FLTR thermal camera (FLTR, USA). Mice were anesthetized after 24 h injection of PBS and C@P, and tumor sites were exposed to 808 nm NIR laser at a power of 1.0 w cm^−2^ for 5 min. An FLTR thermal camera was used to photothermal photos**.**

### Antitumor experiments in H22 tumor models

In antitumor experiments, the H22 tumor model was used to assess efficiency of immune checkpoint and siRNA delivery in synergistic cancer therapy. H22 cells were subcutaneously injected into the right axilla with a density of 1 × 10^6^ cells in 100 μL PBS. Mice were randomly divided into 8 groups to evaluate the therapeutic efficacy (n = 6, C@P 1 mg (Cu)/kg body weight, siRNA 40 ug per mouse; aPD-L1 100ug per mouse): Group I: PBS; Group II: aPD-L1; Group III: C@P; Group IV: C@P + Laser; Group V: C@P + Laser + aPD-L1; Group VI:: CS@P; Group VII:: C CS@P + Laser; Group VIII: CS@P + Laser + aPD-L1. Each mouse was iv injected with 200 µL of corresponding nanoparticle formulation on day 0, 3, 6, and the groups of + laser were then irradiated with an 808 nm laser at 1 W/cm^2^ for 5 min after 24 h injection. During the therapy period, the tumor size and body weight were measured every other day. The tumor volume was calculated using the following formula: V = L × W^2^/2. On the 21st day, three mice were killed and photographed. All major organs ((heart, liver, spleen, lung, kidneys and tumors) are collected and checked by H&E staining. Immunohistochemical staining of PD-L1 and NR2F6 was performed on primary tumors. The survival time of the remaining mice in each group was monitored until the 50th day after the first injection and a survival curve was generated.

### Inhibition of distant tumor growth

H22 cells were subcutaneously injected into the left axilla with a density of 1 × 10^6^ cells in 100 μL PBS to build distant tumor models on Day1. The distant tumor volumes were measured during the period of the experiment.

### Measurement of anti-metastasis effects

H22 cells were subcutaneously injected into the left axilla with a density of 1 × 10^6^ cells in 100 μL PBS on Day-7. On day 0, mice were randomly divided into 4 groups and were treated with PBS, C@P + Laser, CS@P + Laser and CS@P + Laser + aPD-L1, respectively. On day-1, each mouse was injected intravenously with 5 × 10^4^ luc-H22 tumor cells. The IVIS Spectrum (caliper, USA) was used to detect lung metastatic nodules, and the lung was fixed in Bouin's solution to count the nodules.

### Cytokine detection

According to the vendors’ instructions, ELISA kits were used to detected level of TNF-α, IFN-γ, and IL-2.

### Ex vivo analysis of different groups of immune cells

Two tumors, lymph nodes and spleens were cut into small pieces, weighed and homogenized. Homogenates were resuspended in PBS for further flow cytometry. The percentages of CD4^+^ or CD8^+^ T cells in the tumors and spleen were assessed by staining with anti-CD3-FITC, anti-CD4-PE, and anti-CD8-APC antibodies according to the standard protocols. The Tregs were assessed by staining with anti-CD3-FITC, anti-CD4-PE and anti-Foxp3-Pacific Blue antibodies. The frequency of matured DCs in the lymph nodes was detected by staining with anti-CD80-PE and anti-CD86-APC antibodies in the gated CD11^+^ cells. As for memory T cells, the cells gated by CD3^+^CD8^+^CD44^+^CD62L^+^ and CD3^+^CD8^+^CD44^+^CD62L^−^ were distinguished as central memory T cells and effector memory T cells, respectively.

### Statistical analysis

Quantitative data were presented as mean ± SD. The results were analyzed using two-tailed Student’s t tests between two groups or One-way analysis for multiple-groups. *P* < 0.05 was set as statistic significant.

## Results and discussion

### Construction and physicochemical features of the pH-responsive charge-reverse CS@P

**Fig. 2:**
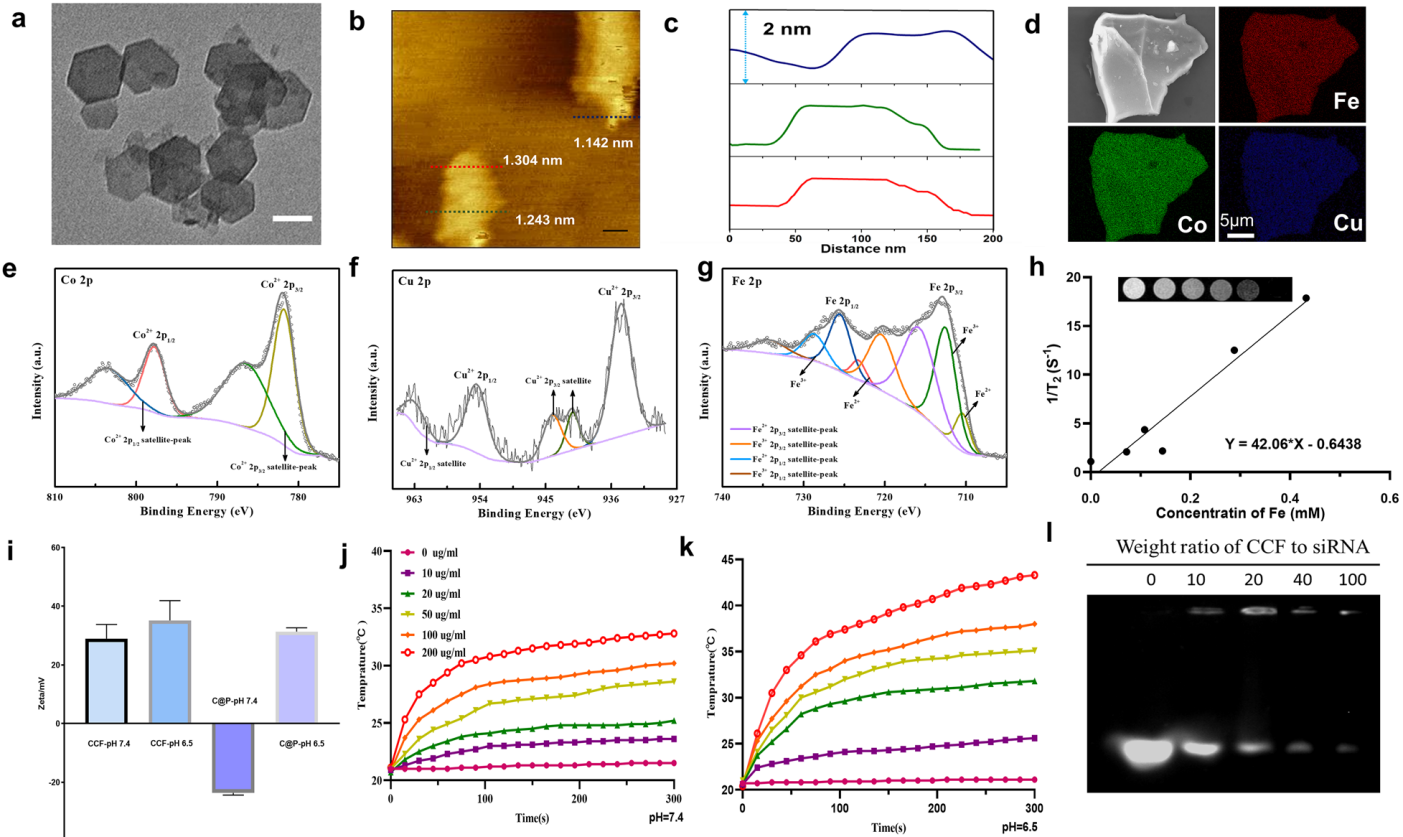
Characterizations of CS@P. **a** HRTEM image of the CCF-LDHs. Scale bar = 50 nm. **b** AFM image and **c** represented the thickness of CCF-LDHs monolayer nanosheets. **d** EDS mapping of the CCF-LDHs nanosheets. XPS spectra of **e** Co 2*p*, **f** Cu 2*p* and **g** Fe 2*p*. **h** T2 relaxation versus Fe concentration of CCF-LDHs and inset shows the corresponding T2-weighted images. **i** Zeta potential of CCF-LDHs and C@P in pH 7.4 and 6.5 media. Error bars stand for ± SD (n = 3). Photothermal curves of C@P dispersion at different concentrations irradiated by 880 nm laser at 1.0 W/cm^2^ for 5 min in pH = 7.4 (**j**) and 6.5 (**k**), respectively. **l** siRNA loading analysis at different w/w ratios of CCF-LDHs to siRNA

The fabrication of CS@P is described in Fig. 1a. The CCF-LDHs monolayer nanosheets were prepared using a facile bottom-up approach reported in previous studies [39]. The hexagonal shape of the CCF-LDHs with an average size of 78 nm was clearly observed in the TEM image (Fig. 2a). The CCF-LDHs exhibited hydrodynamic diameters of ~ 84 nm, ~ 99 nm, and ~ 126 nm in water, PBS and RPMI 1640 medium, respectively (Additional file 1: Fig. S1). Moreover, we observed no significant changes in the hydrodynamic dimensions of the nanosheets over the course of a week, indicating that the CCF-LDHs were highly stable (Additional file 1: Fig. S2). Atomic force microscopy (AFM) images showed that the thickness of the CCF-LDHs was ∼1.1 nm (Fig. 2b and c), revealing a single-layer structure. The X-ray diffraction (XRD) pattern of the CCF-LDHs was displayed in Additional file 1: Fig. S3. As shown, a typical sequence corresponding to planes (003), (006), and (009) illustrated the lamellar structure of the CCF-LDHs. According to inductively coupled plasma–mass spectrometry (ICP–MS) analysis, the Co/Cu/Fe molar ratio in the CCF-LDH nanosheets was 1.94:0.45:1, which is close to the feed ratio. In addition, energy-dispersive spectroscopy (EDS) was performed to confirm the elemental compositions and distributions of the materials. The Elemental mapping (Fig. 2d) and EDS images of the CCF-LDHs (Additional file 1: Fig. S4) revealed the distributions of Co, Cu, and Fe in the nanostructure.

Furthermore, we adopted XPS to study the chemical state of the CCF-LDHs. In the Co 2*p* spectrum (Fig. 2e), there were two main peaks at 781.78 eV (Co 2*p*_3/2_) and 797.75 eV (Co 2*p*_1/2_) and two satellite peaks (785.47 eV and 803.39 eV), which indicated a high-spin Co^2+^ state. In the Cu 2*p* spectrum (Fig. 2f), the characteristic peaks of Cu 2*p* (Cu^2+^) were found at both 934.67 eV (2*p*_3/2_) and 954.46 eV (2*p*_1/2_). As shown in Fig. 2g, mixed-valence Fe^2+^ and Fe^3+^ states appeared in the nanosheets with an Fe^2+^/Fe^3+^ ratio of 0.24. Moreover, Co^2+^ and Fe^3+^ with unpaired 3d electrons can act as T2 contrast agents [40], and the element Cu(II) in the host layer is a T1 contrast agent [41]. This combination of elements endowed the CCF-LDHs with potential T2-weighted MRI ability that favors tumor treatment guidance and monitoring (Fig. 2h). On the other hand, the T1-weighted MRI scan was not acceptable, possibly due to the low Cu to Fe concentration ratio.

To endow CCF-LDHs with charge reversibility in response to the acidic tumor extracellular environment, pH-responsive charge-reversal PEG-DMMA was incorporated to shield the nanosheets [38]. PEG-DMMA was synthesized through the reaction between the amines in 6NH2-PEG and the anhydride in DMMA. ^1^H NMR spectroscopy was used to confirm the results. The signals at 3.50–3.52 ppm and 1.89 ppm were assigned to the methylene protons of PEG and the methyl group of DMMA, respectively (Additional file 1: Fig. S5). The positively charged CCF-LDHs were then shielded with the PEG-DMMA polymer through electrostatic interactions to form C@P***. ***The constitution of C@P was confirmed by FT-IR spectroscopy (Additional file 1: Fig. S6). Moreover, the average size of C@P was ~ 100 nm (Additional file 1: Fig. S7). As shown in Fig. 2i, CCF-LDHs exhibited non-charge-reversal properties with a stable positive charge in PBS at both pH 7.4 and 6.5, whereas the surface charge of C@P was changed from − 23.6 mV to 31.3 mV after incubation in pH 6.5 PBS at 37 °C for 60 min. These changes were due to the negatively charged PEG-DMMA reversing to a positive charge and detaching from the positively charged CCF-LDHs via electrostatic repulsion. Moreover, the photothermal effect of C@P at different concentrations and laser power densities was investigated. As show in Fig. 2j and k, at pH = 7.4 and 6.5, the temperature increment (ΔT) could reach 11.7 °C and 22.9 °C at a concentration of 200 µg mL^−1^, respectively (808 nm laser, 1.0 W cm^−2^, 300 s). These results may be attributed to the greater number of defects generated by the lower pH buffer, which increased the number of free charge carriers, resulted in localized surface plasmon resonances and converted electromagnetic (light) energy to thermal energy[24, 42]. It was also found that the photothermal conversion ability of C@P was laser power-dependent (Additional file 1: Fig. S8). Thermal infrared images of C@P in tubes were acquired (Additional file 1: Fig. S9). Gel electrophoresis was used to assess the capacity of the CCF-LDHs to complex siRNA, and a weight ratio of 100 was determined to be sufficient to maintain high encapsulation efficiency (Fig. 2l). There were no clear changes in the size or zeta potential of CCF-LDHs-siRNA were detected after siRNA complexing (Additional file 1: Figs. S10 and S11). The stability of CCF-LDHs in 1640 without fetal bovine serum (FBS) was good, while apparent aggregation was observed in 1640 with 10% FBS (Additional file 1: Fig. S12, bottom left). After PEG-DMMA modification, the colloidal stability of LDH nanoparticles in 1640 with FBS could be well maintained (Additional file 1: Fig. S12).

### H22 and T-cell dual cellular uptake and gene silencing

**Fig. 3:**
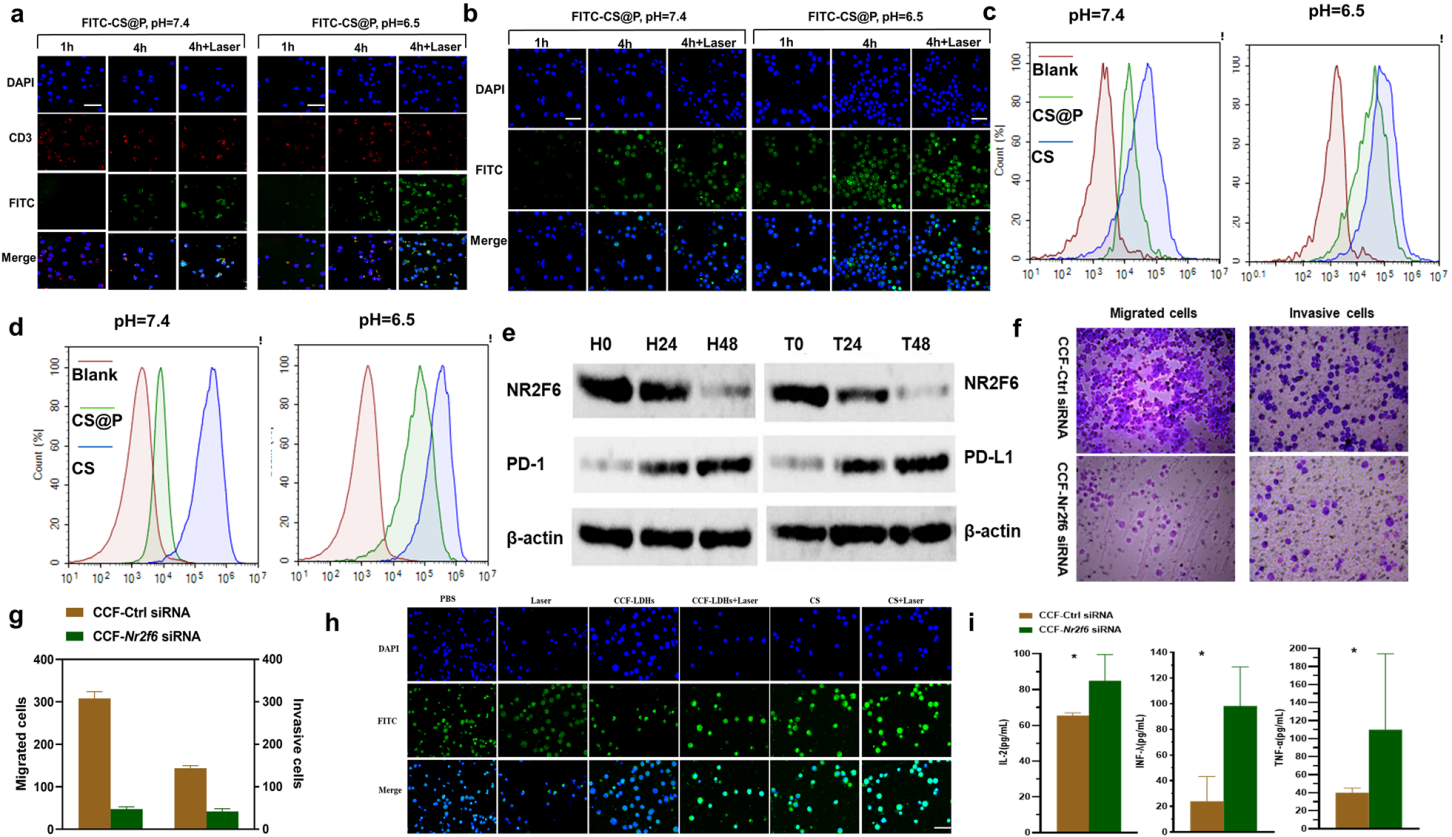
H22 and T cell dual cellular uptake and gene silencing. CLSM images of **a** T cells and **b** H22 tumor cells treated with CS@P in media at pH 7.4 or 6.5, respectively. Scale bar: 50 µm. Flow cytometry images of cellular uptake of FITC-labeled nanoparticles by **c** T cells and **d** H22 tumor cells at pH 7.4 and pH 6.5 conditions for 4 h. **e** Western blot analysis of the indicated proteins in T cells (left) and H22 tumor cells (right) 48 h after the incubated with CS. **f**, **g** Transwell results of the effect of NR2F6 on migration (left) and invasion (right). **h** CLSM images of PD-L1 expression in H22 cell after the indicated treatments. **i** Cytokine levels in the supernatant on 48 h after the indicated treatments (n = 3)

To evaluate the effect of CS@P on pH-dependent cellular uptake, CLSM and flow cytometry analyses were performed. FITC-labeled CS@P was incubated with H22 cells and T cells to examine their internalization in media for 4 h at pH 7.4 or 6.5, respectively. The CLSM images revealed stronger green fluorescence (FITC green emission) at pH 6.5 than at pH 7.4, indicating enhanced cellular uptake in both T cells and H22 cells (Fig. 3a and b). Due to the pH-responsive charge-reversal characteristics of the nanoparticles, the positively charged CCF-LDHs have a far higher affinity for the negatively charged cell membrane, resulting in increased cellular absorption. Meanwhile, local PTT generated by CS@P exhibited a stronger green fluorescence, indicating enhanced cellular uptake, which could be due to an increased pathological permeability impact. As shown in Fig. 3c, in T cells, the cellular uptake of CS was almost identical at pH 7.4 and pH 6.5. Compared to pH 7.4, CS@P showed greatly enhanced cellular uptake at pH 6.5, which was consistent with the CLSM analysis. Figure 3d depicts similar results in H22 cells. As it shown in Additional file 1: Fig. S13, the fluorescence of the T cells and H22 cytoplasm 4 h post-transfection was bright while the nuclei were relatively dark suggesting that CS@P mostly accumulated in the cytoplasm.

NR2F6 is a mechanistically independent negative regulator in effector T cells that governs the amplitude of anticancer immunity [20, 23]. In addition, a previous study found that decreased NR2F6 expression suppressed HCC cell migration and invasion [22]. Next, we examined the effect of NR2F6 gene silencing in H22 tumor cells and T cells, and T cells were stimulated by CD3/CD28 in vitro for 48 h in advance. Western blot analysis was applied to determine the level of the target protein after siRNA knockdown, and the results showed that the CS nanoparticles could significantly reduce NR2F6 protein expression in both H22 and T cells (Fig. 3e). In addition, PD-1 and PD-L1 expression was increased in H22 and T cells (Fig. 3e), suggesting that the loss of NR2F6 expression likely enhanced the activity of established PD-1/PD-L1 checkpoint blockade [23]. The transwell assay results demonstrated that NR2F6 knockdown suppressed the migration and invasion of H22 cells (Fig. 3f and g).

Next, we examined the impact of mild PTT in our combination strategy. H22 cells were irradiated with an 808 nm laser for 5 min at 4 h after the corresponding processing, and CLSM was used after 48 h of incubation. Green fluorescence of FITC was strongest in the CS + Laser group, which indicated increasing PD-L1 expression of H22 cells (Fig. 3h and Additional file 1: Fig. S14), due to upregulation of self-protection proteins PD-L1 by mild warmth and genetic NR2F6 ablation. Mild heating (42–45 °C) combined with combination therapy significantly enhanced tumor sensitivity to immune checkpoint suppression by significantly increasing PD-L1 expression. We proceeded to explore the expression levels of cytokines that potently favor tumor rejection, such as IL-2, TNF-α and IFN-γ. The ELISA results are shown in Fig. 3i and support the suitability of combination therapy with aPD-L1, which contributed to enhancing the synergistic effect when combined with established checkpoint blockade.

### In vitro therapeutic effects of NR2F6 knockdown and PTT

**Fig. 4:**
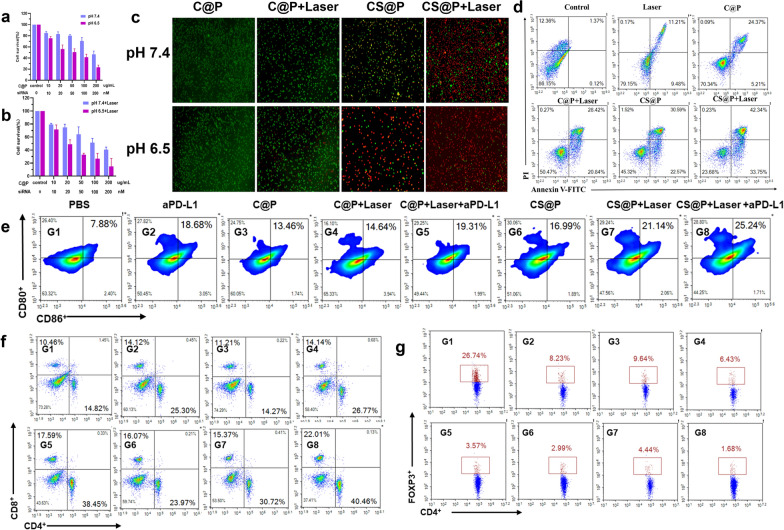
In vitro immune responses mediated by CS@P with laser irradiation. In vitro cell viability of H22 tumor cells incubated with CS@P for 24 h without (**a**) and with (**b**) laser irradiation (300 s, 1 W cm^−2^). Error bars stand for ± SD (n = 3). **c** Calcein AM/PI staining result of H22 tumor cells after the indicated treatments (C@P concentration: 100 μg mL^−1^, siRNA 100 nM). **d** Flow cytometry *analysis* of apoptosis levels in H22 cells after treatment with PBS, laser, C@P, C@P + laser, CS@P, or CS@P + laser (100 μg/mL C@P, 100 nM siRNA) for 24 h with 808 nm laser irradiation (300 s, 1 W cm^−2^) or not. **e** The percentages of mature DCs (CD11c^+^CD86^+^CD80^+^) through flow cytometry after indicated treatments in vitro DCs/H22 co-culture system. Error bars stand for ± SD (n = 3). **f** The flow cytometry plots and proportions of CD4^+^ and CD8^+^ T cells in vitro T lymphocytes/DCs/H22 cells (50:10:1) triple co-culture system (gated on the CD3^+^). Error bars stand for ± SD (n = 3). **g** The Treg flow cytometry analysis and frequencies in vitro T lymphocytes/DCs/H22 cells (50:10:1) triple co-culture system. Error bars stand for ± SD (n = 3)

To show the therapeutic effects of PTT, H22 tumor cells were incubated with C@P and treated with 808 nm laser irradiation. Without 808 nm NIR laser irradiation, C@P showed no obvious cytotoxicity at either pH 7.4 or 6.5 (Additional file 1: Fig. S15), which affirmed its excellent biocompatibility. As expected, upon 808 nm laser irradiation, the mild increase in temperature showed limited cytotoxicity in neither pH 7.4 (cell viability ≈ 70–90%) nor 6.5 (cell viability ≈ 60–80%) culture medium (Additional file 1: Fig. S16). The viability of cells treated with CS@P in pH 7.4 and 6.5 medium decreased in a dose-dependent manner (Fig. 4a). Compared with pH 7.4 medium (cell viability 71%), CS@P treatment showed higher cytotoxicity in pH 6.5 medium at [C@P] = 100 µg mL^−1^ and [siRNA] = 100 nM (cell viability 41%), which further demonstrated that the pH-responsive charge-reversal characteristics enhanced cellular uptake. To further investigate the in vitro synergy between siRNA and PTT, 808 nm NIR laser irradiation and *Nr2f6* siRNA were combined to treat H22 cells using CS@P. In sharp contrast, CS@P treatment combined with laser irradiation showed higher cytotoxicity in cancer cells, particularly at pH 6.5 (Fig. 4b). Specifically, the cell viability decreased to 27% in pH 6.5 medium at [CS@P] = 100 µg mL^−1^ and [siRNA] = 100 nM upon 808 nm laser irradiation (deep purple bar, Fig. 4b), whereas the cell viability was 41% under the same treatment conditions without irradiation (deep purple bar, Fig. 4a). These results demonstrated the synergy between siRNA and PTT, which may be ascribed to the increased membrane permeability caused by PTT, driving additional cellular uptake [43]. The confocal fluorescence images of HCC tumor cells stained with a calcein AM/propidium iodide (PI) kit allowed visualization of the distribution of viable and dead cells, which was found to be consistent with the CCK-8 results (Fig. 4c). As shown in Fig. 4d, a cell apoptosis assay in H22 cells further confirmed the potent cytotoxicity of CS@P treatment combined with 808 nm laser irradiation.

Several investigations have verified that PTT, as a new approach for precision cancer therapy, could induce ICD followed by tumor-associated antigen release, which could trigger further immune responses [44, 45]. Based on the above-described experimental results, CS@P in our study acted as a “sensitizer” for established PD-1/PD-L1 checkpoint blockade. Therefore, we wondered whether combining CS@P with PTT and aPD-L1 would enhance downstream immunological responses in vitro. To examine the maturation of DCs, we established a DC/H22 coculture system with the corresponding treatments. We detected a significant increase in mature DC (CD11^+^CD80^+^CD86^+^) numbers from 7.88% to 19.31% and 25.24% in the CP + Laser + aPD-L1 and CS@P + Laser + aPD-L1 groups, respectively, compared with those in the other groups (Fig. 4e), suggesting that the combination of PTT and aPD-L1 treatment stimulated the maturation of DCs. Then, we established a T lymphocyte/DCs/H22 cells (50:10:1) triple co-culture system to examine the effects on the activation of T cells. T lymphocytes were previous incubated with CCF-*Nr2f6* siRNA and CCF-negative siRNA nanoparticles for 48 h. The three types of cells were treated with H22 cell lysates that had previously received various treatments. Helper T lymphocytes (HTLs) (CD3^+^CD4^+^) play critical roles in regulating adaptive immunity, and cytotoxic T lymphocytes (CTLs) (CD3^+^ CD8^+^) can directly kill targeted tumor cells.

As shown in the flow cytometry images (Fig. 4f), the numbers of HTLs and CTLs were markedly higher in the CP + Laser + aPD-L1 and CS@P + Laser + aPD-L1 treatment groups. The percentages of HTLs and CTLs reached as high as 40.46% and 22.01% after treatment with the combination of CS@P, laser and aPD-L1, respectively. As a control, the percentages of HTLs and CTLs in the PBS group were only 14.82% and 10.46%, respectively. In contrast, the number of regulatory T cells (Tregs) (CD4^+^Foxp3^+^), which can hamper effective antitumor immunological responses, decreased substantially to 1.68% in the CS@P + Laser + aPD-L1 treatment group compared with 26.74% in the PBS group (Fig. 4g). These results supported that CS@P plus laser irradiation and aPD-L1 could stimulate the maturation of DCs and efficiently promote antitumor immunological responses.

### In vivo nanoparticle MRI capacity and biodistribution pattern

Encouraged by the exciting in vitro results, we assessed the biodistribution and real-time imaging of CS@P in H22 tumor-bearing mice. Fluorescence imaging was used to demonstrate the biodistribution of ICG-labeled CS@P at different time points. Compared with the CS nanoparticles, CS@P could effectively accumulate in the tumor regions, and the fluorescence intensity peaked at 24 h postinjection (Additional file 1: Fig. S17), which may be attributed to the long blood circulation time of the nanoparticles and enhanced permeability and retention (EPR) effect [46]. Organs and tumors were harvested at 48 h postinjection for ex vivo fluorescence imaging (Additional file 1: Fig. S18). We further investigated the biodistribution and enhanced tumor accumulation by MRI. Since Co(II) and Fe(III) with unpaired 3d electrons are T2-weighted MRI contrast agents [40], and the transition metal Cu(II) can shorten the T1 relaxation time of protons in magnetic fields [47], CS@P was endowed with potential T1/T2 MRI ability. As shown in Additional file 1: Fig. S19, the T1/T2-weighted MR signal brightness within the tumor sites changed in a time-dependent manner. In vivo T1-weighted MRI showed that the brightness enhanced gradually after injection, and the maximum value was observed at 24 h post injection, which was in line with the fluorescence imaging results. Similarly, the T2-MRI signal intensity increased until 24 h post injection and then gradually declined until 48 h. These results confirmed that CS@P could effectively accumulate in the tumor region and showed promising ability as a dual-modal imaging agent for T1/T2 MRI, which can subsequently guide combined tumor therapy.

### In vivo antitumor evaluation in an H22 tumor model

**Fig. 5:**
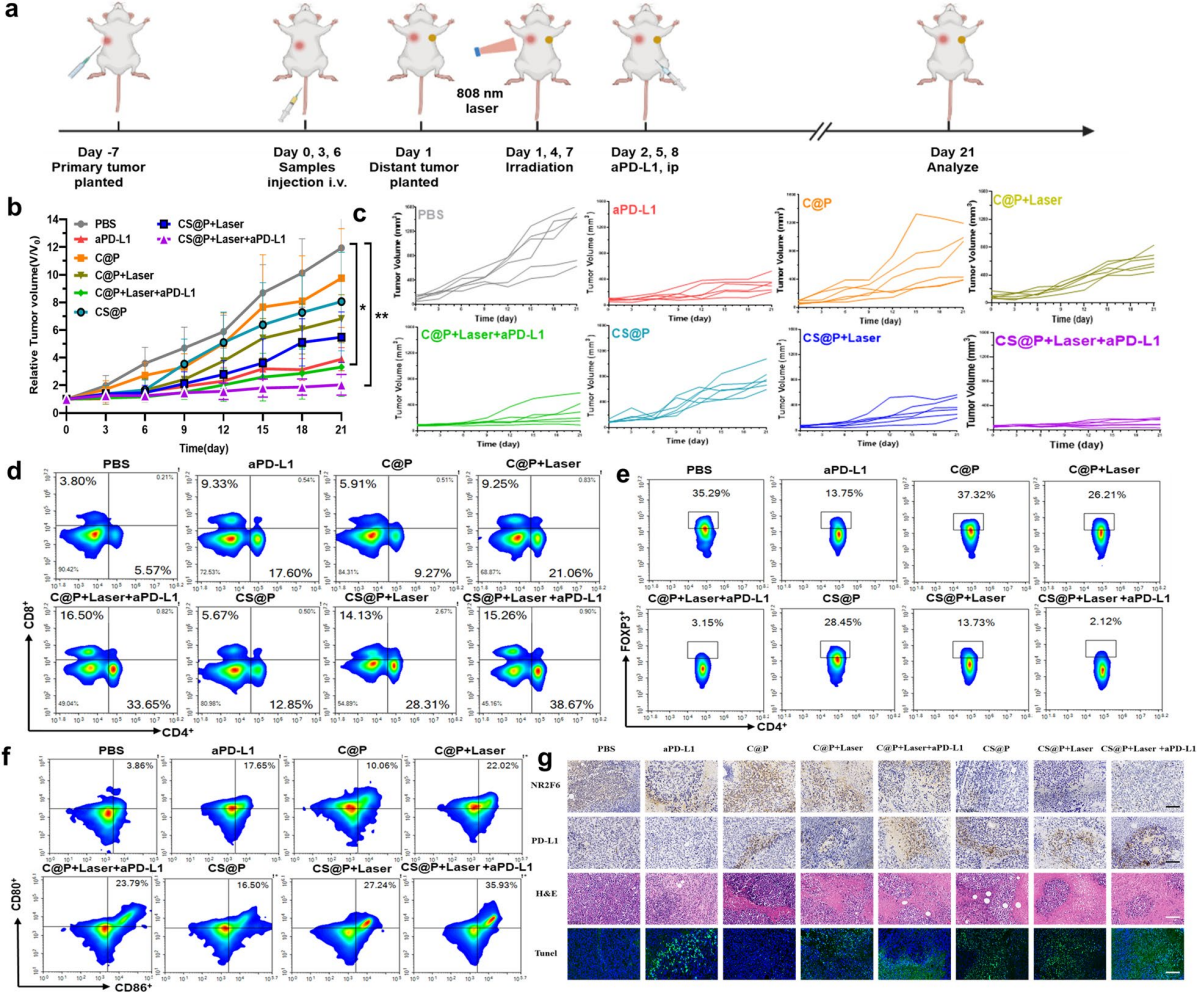
Immune response of CS@P together with laser and PD-L1 in vivo. **a** Schematic diagram of the model and therapeutic schedule of primary and distant H22 tumor model. **b** Primary tumor growth tendency of H22 tumor-bearing mice with various treatments. Tumor sizes were normalized to initial sizes. Error bars stand for ± SD (n = 6). **c** Primary tumor growth curves in the H22 tumor-bearing BALB/c mice (n = 6). **d** Representative flow cytometry plots showing different groups of T cells in primary tumors (gated on CD3^+^ T cells) after indicated treatments. **e** The Treg flow cytometry analysis in primary tumors after various treatments. **f** Representative flow cytometry plots showing matured DC cells in primary tumors after various treatments. **g** Immunohistochemical staining of NR2F6 and PD-L1 in primary tumor sections (top). H&E and TUNEL examination of tumor sections (bottom). Scale bar: 100 µm*.* *P < 0.05, **P < 0.01, and ***P < 0.001

A H22 mouse tumor model was used to assess the antitumor effects of our proposed siRNA-assisted assembly strategy. To evaluate the photothermal effect in vivo, the temperature in tumor site with the indicated treatment was recorded by an IR thermal camera (Additional file 1: Fig. S20). Mice were anesthetized after 24 h injection of PBS and C@P, and tumor sites were exposed to 808 nm NIR laser at a power of 1.0 w cm^−2^ for 5 min. Under irradiation, the temperature of mice treated with C@P was increased from ∼32 °C to ∼45 °C. It revealed that C@P can induce mild PTT in vivo efficiently. To further assess antitumor evaluation in vivo, H22 tumor-bearing mice were randomly allocated into one of eight (n = 6) groups. When the tumor volumes reached ∼100 mm^3^ on day 0, each group was treated with i.v. injection of CCF-LDHs (1 mg of Cu kg^−1^ body weight) and/or siRNA (40 µg per mouse) (Fig. 5a) as follows: Group I: PBS; Group II: aPD-L1; Group III: C@P; Group IV: C@P + Laser; Group V: C@P + Laser + aPD-L1; Group VI: CS@P; Group VII: CS@P + Laser; and Group VIII: CS@P + Laser + aPD-L1. NIR laser irradiation (808 nm, 1 W cm^−2^, 5 min, 42–45 °C) was applied to the mice three times on days 1, 4 and 7 at 24 h post injection according to the pattern shown in Fig. 5a. The dose of aPD-L1 in each group was 100 μg per mouse. As a control, the group treated with PBS was negligibly inhibited (Fig. 5b and c).

Administration of the combination therapy in to Group VIII, primary tumor growth was dramatically inhibited within 21 days, which indicated the excellent synergistic therapeutic effect of *Nr2f6* siRNA knockdown, mild PTT and aPD-L1. However, the tumors in the CS@P group exhibited moderate growth, suggesting that *Nr2f6* siRNA knockdown alone had a limited therapeutic effect on primary tumor growth (Fig. 5b). These results are in line with the fact that PD-L1 expression is upregulated with *Nr2f6* siRNA knockdown, suggesting potential self-protection and resistance mechanisms. The residual tumors were excised on day 21 post injection, and the digital images of the mice in each group visually demonstrated the antitumor effects of the different treatments (Additional file 1: Fig. S21).

To verify the enhanced synergistic antitumor effect induced by CS@P-mediated PTT in combination with aPD-L1 therapy, the numbers of immune cells in the primary tumors were measured on day 10, as well as immune cell cytokine contents. Combined with laser irradiation and PD-L1 blockade therapy, the C@P and CS@P treatments showed much greater efficacy than monotherapy in terms of activating T lymphocytes (Fig. 5d). Compared with the PBS group, we found significantly increased numbers of CD4^+^ and CD8^+^ tumor-infiltrating T cells in the mice treated with CS@P-mediated PTT in combination with aPD-L1 therapy. The percentages of CD4^+^ HTLs and CD8^+^ CTLs in the CS@P + Laser + aPD-L1 group were 6.9- and 4.0-fold higher than those in the PBS group, respectively. In contrast, the number of CD4^+^Foxp3^+^ immune-suppressive Treg cells was significantly decreased in this group (Fig. 5e). As reported previously, PTT can not only inhibit tumor growth but also release tumor antigens to induce the maturation of DCs, which plays an important role in initiating immune responses. Here, we investigated the expression of the costimulatory molecules CD80 and CD86 (gated on CD11c^+^) in the nearby tumor-draining lymph nodes after treatment to evaluate DC activation. As shown in Fig. 5f, after treatment with CS@P-mediated PTT in combination with aPD-L1 therapy, the percentage of mature DCs increased to ∼35.16%, which was higher than that in all the other groups. Immunohistochemical staining of primary tumor sections confirmed that the expression level of NR2F6 decreased and PD-L1 was notably upregulated in mice after CS@P treatment (Fig. 5g). In addition, primary tumors were further stained with hematoxylin and eosin (H&E) and TUNEL, showing evident tumor cell death in mice treated with CS@P-mediated PTT and aPD-L1. The above results underscore the advanced synergistic efficacy of our siRNA-assisted assembly strategy to reverse the immunosuppressive TME by augmenting the expression of PD-L1 and PD-L1 depletion.

### Inhibiting distant tumor growth

**Fig. 6:**
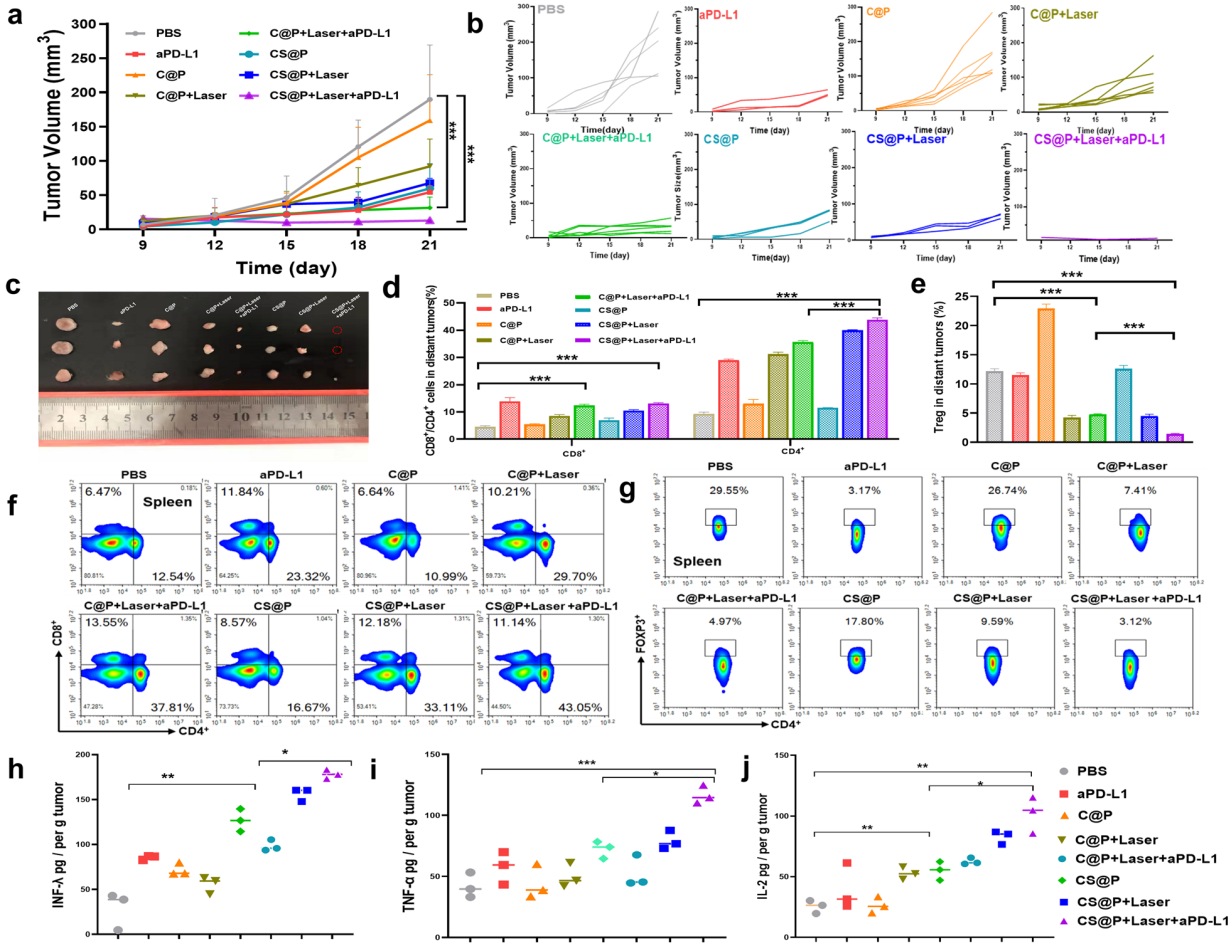
Abscopal effect of CS@P combined with NIR irradiation and aPD-L1. **a** Distant tumor growth tendency of H22 tumor-bearing mice with various treatments. Tumor sizes were normalized to initial sizes. Error bars stand for ± SD (n = 6). **b** Distant tumor growth curves in the H22 tumor-bearing BALB/c mice (n = 6). **c** Images of the distant tumor harvested on day 21. **d** The amount of CD4^+^ and CD8^+^ T cells in distant tumors detected upon various treatments detected by flow cytometry (gated on the CD3^+^). Data represented mean ± SD (n = 3). **e** The amount of Treg cells in distant tumors upon various treatments detected by flow cytometry Data represent mean ± SD (n = 3). **f** Frequency of the CD4^+^ and CD8^+^ T cells in spleens after indicated treatments. **g** Frequency of the Treg cells in spleens after indicated treatments. The levels of IFN-γ (**h**), TNF-α (**i**), and IL-2 (**j**) in the primary tumor after the various treatments (n = 3), expressed as the concentration per gram of tumor (pg/per g tumor). *P < 0.05, **P < 0.01, and ***P < 0.001

To investigate whether the enhanced immune response induced by CS@P-mediated PTT in combination with aPD-L1 therapy could inhibit the growth of an untreated distant tumor, we established a dual-tumor model by injecting H22 tumor cells into the mouse flanks opposite that of the primary tumor on day 1 (Fig. 5a). After local primary tumor treatment, we measured the growth of the distant tumors with a caliper every three days (Fig. 6a and b). Strikingly, CS@P-mediated PTT together with PD-L1 depletion restricted the growth of the secondary tumors, which affirmed the enhanced systemic immunity (Fig. 6c). However, treatment without aPD-L1 therapy, i.e., CS@P or CS@P + Laser, showed a moderate effect on distant tumor growth, suggesting that systemic immunity may be limited in the immunosuppressive TME of the secondary tumors in the absence of combination therapy. We further investigated the intratumoral infiltration of activated lymphocytes by flow cytometry. Markedly elevated frequencies of HTLs and CTLs in the distant tumors were observed in the combined therapy group (Fig. 6d). The frequencies of CD4^+^ HTLs and CD8^+^ CTLs in the CS@P + Laser + aPD-L1 group were ~ 4.7- and ~ 3.1-fold higher than those in PBS, respectively. Moreover, compared to that in the PBS group, the level of Tregs in the CS@P + Laser + aPD-L1 group was markedly lower, confirming the reversal of immunosuppression in the distal tumors (Fig. 6e). Correspondingly, the population of CD4^+^ HTLs (~ 43.05%) and CD8^+^ CTLs (11.14%) in the spleen displayed a significant increase after CS@P + Laser + aPD-L1 treatment, whereas the Treg population decreased to 3.12% (Fig. 6f and g). The levels of IL-2, TNF-α, and IFN-γ, which are critical biomarkers for altering immune responses in the TME, in the primary tumor were significantly increased in the CS@P + Laser + aPD-L1 group (Fig. 6h–j). Compared with the PBS group, the survival time of the mice treated with CS@P plus a laser and aPD-L1 increased by approximately 12 days, affirming that the siRNA-assisted assembly strategy together with PTT and aPD-L1 effectively inhibited primary and distant tumor growth and increased the survival rates (Additional file 1: Fig. S22). Additionally, the mouse body weights exhibited negligible changes in all groups (Additional file 1: Fig. S23), and H&E staining images of the major organs (heart, liver, spleen, lung, and kidney) confirmed that there was no clear inflammatory infiltration or damage, indicating the biosafety of all formulations (Additional file 1: Fig. S24).

### Evaluation of the in vivo antimetastatic effects

**Fig. 7:**
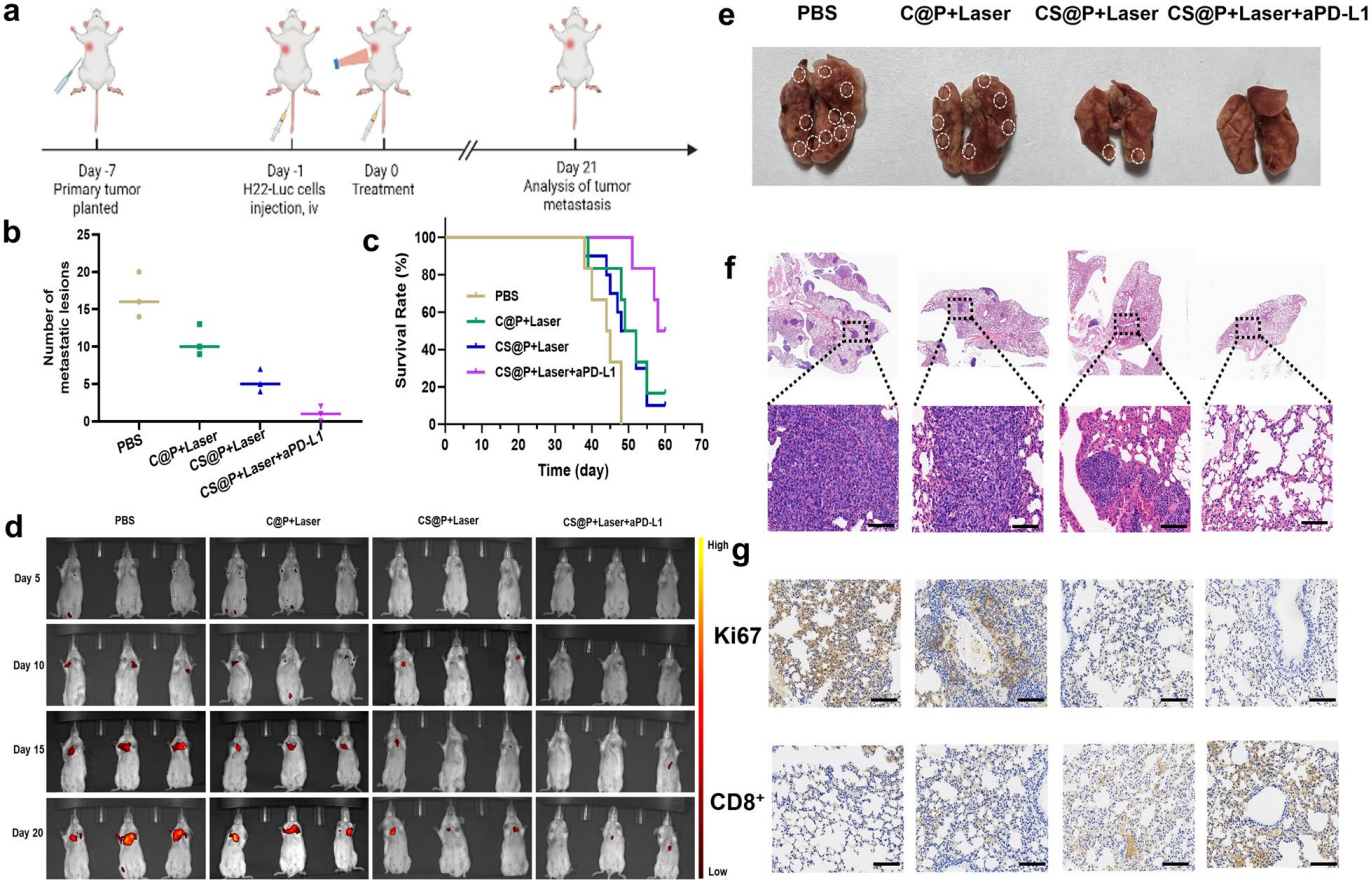
Anti-metastasis effect of CS@P combined with NIR irradiation and aPD-L1. **a** Schematic diagram of the model and therapeutic schedule of metastatic H22 tumor model. **b** Number of pulmonary metastatic lesions after different treatments (n = 3). **c** Survival curves of H22 tumor-bearing mice after different treatments (n = 6). **d** In vivo bioluminescence images of the lungs on day 5, 10, 15 and 20 (n = 3). **e** Representative photographs of metastatic nodules after different treatments. **f** H&E staining of harvested lung tissues on day 21. Scale bars: 100 μm. **g** Immunohistochemical staining of Ki67 and CD8^+^ of isolated lung sections. Scale bars: 100 μm

Encouraged by the potent performance of combination therapy to inhibit the growth of both primary and distal tumors, we further investigated lung metastasis in the different groups after the appropriate treatments. In our experiment, we established a primary tumor model in the right armpit on day-7. When the primary tumor volume reached approximately 75 ~ 100 mm^3^ on day-1, H22-Luc tumor cells were further i.v. injected into the mice. The primary tumors in the PBS, C@P plus laser, CS@P plus laser, CS@P plus laser and aPD-L1 groups received direct treatment (Fig. 7a). It was found that CS@P plus a laser and aPD-L1 not only restrained the growth of the primary tumors but also suppressed lung metastatic lesions (Fig. 7b). Compared with the PBS group, the long-term survival rate was significantly increased to 50% in mice treated with CS@P plus a laser and aPD-L1, suggesting that the synergistic antitumor effect suppressed distant metastasis to prolong the lifespan of the tumor-bearing mice (Fig. 7c). In addition, the bioluminescence of the H22 cells in the mice demonstrated a synergistic effect to inhibit lung metastasis (Fig. 7d), as affirmed by digital photographs and H&E staining (Fig. 7e and f). Anti-Ki67 staining further indicated the considerably inhibited proliferation of Luc-H22 cells (Fig. 7g). As shown in Fig. 7g, CS@P plus a laser and aPD-L1 therapy induced more infiltration of CD8^+^ CTLs into the lung metastatic tumors.

To understand the mechanism of the antimetastatic effect triggered by CS@P-mediated PTT in combination with aPD-L1 therapy, we further studied adaptive immunity establishment on day 21. Immunological memory T cells are classified into distinct T-cell subsets: central memory T cells (T_CM_ cells; efficiently stimulate DCs, generate effector cells and help B cells) and effector memory T cells (T_EM_ cells; induce immediate inflammatory reactions or cytotoxicity)[48, 49]. Therefore, we analyzed the proportions of T_EM_ cells (CD3^+^CD8^+^CD62L^−^CD44^+^) in the spleens and tumor-draining lymph nodes. It was found that the T_EM_ cell frequency significantly increased in mice treated with CS@P plus laser irradiation and aPD-L1 in both the spleens and lymph nodes (Additional file 1: Fig. S25a and S25b), which suggested an enhanced immune memory effect that may be attributed to the inhibition of cancer metastasis.

## Discussion

Constructed over decades, immunotherapy has played a promising role and has shown tremendous prospects in cancer treatments, which aims to harness the inherent immunological system to kill tumor cells [50]. As one of the most widely used immunotherapeutic approaches, PD-1/PD-L1 pathway blockade has exerted significant effects against HCC. Although promising, only a small portion of HCC patients benefit from this therapy [9], highlighting the need to improve the anti-tumor immunotherapy responses of established PD-1/PD-L1 checkpoint blockade. In view of the dynamic complicated TME, combining a synergistic immune checkpoint with PD-L1 checkpoint blockade could increase the response rates of HCC patients. Instead of killing tumor cells directly, mild PTT was proposed as an aid in cancer treatment. Theoretically, mild heating could overcome the immunosuppressive TME and potentiate the immune response. In this study, we explored a therapeutic strategy combining the intracellular immune checkpoint NR2F6 and the surface immune checkpoint NR2F6 for HCC treatment. We reported a synergistic therapeutic strategy involving dual immune checkpoint blockade inside and on the surface of immune cells, i.e., PD-1/PD-L1 blockage/genetic NR2F6 ablation, combined with mild PTT. This combination treatment can not only successfully eradicate primary H22 tumors in a mouse model but also effectively prevent tumor metastasis.

Immune surveillance and immune elimination are essential for effective cancer control.

The expression of PD-L1 on surface of tumor cells “protects” tumor cells from immune defense and induces “immune evasion”. Additionally, the presence of NR2F6 limits the activation of effector T cells. We demonstrated a combined strategy to strengthen the weapon (T cells) and neutralize the self-protection of tumor cells. Presently, siRNA has emerged as a promising therapeutic approach, which can silence targeted genes and subsequently down-regulate gene expression [51]. However, the delivery of “naked” siRNA is invalid due to its high negative charges and susceptibility to degradation in vivo. In this study, *Nr2f6* siRNA was loaded into LDH nanosheets, and PEG-DMMA was incorporated to shield the nanosheets. Under the acidic TME, the constructed nanoparticle CS@P sequentially detached the “camouflage” and reversed to a positive charge. The re-exposed hexagonal CCF-LDHs loaded with *Nr2f6* siRNA were efficiently internalized and triggered *Nr2f6* siRNA release to down-regulate NR2F6 expression in both immune and H22 cells, which blocked its dual pro-tumor activity [21, 22]. We observed that genetic NR2F6 ablation alone could not eradicate primary tumors due to the upregulation of PD-L1 expression after the knockdown of *Nr2f6* siRNA (Figs. 3h and 5b). Furthermore, mild heating generated by CCF-LDHs also evoked potential self-protection and upregulated PD-L1 expression. These findings supported the reasonable combination of genetic NR2F6 ablation and mild PTT with aPD-L1.

Studies have shown that the complicated immunosuppressive TME limits the efficiency of PD-1/PD-L1 blockade. The localized, mild heating produced by Cu-doped LDHs induces ICD, activates DCs, and recruits TILs. We hypothesized that three combinations of PTT, genetic NR2F6 ablation, and PD-L1 blockade could induce powerful synergistic antitumor immunity. We observed that the combination approaches could reverse the “cold” state into a “hot” state and remodel the immunosuppressive TME in H22 tumor models. According to our results, the proportions of Tregs in primary and distant tumors were significantly decreased in the CS@P + Laser + aPD-L1 group (Figs. 5e and 6e). Since the matured DCs play an important role in stimulating T-cell responses, we evaluated DC activation in the nearby tumor-draining lymph nodes after the indicated treatments. Compared with PBS group, the percentage of mature DCs was 9.3-fold higher (Fig. 5f). The results further underscored the promising synergistic efficacy in immune-suppressive TME reversion of our united immunotherapy strategy. Ideal clinical cancer immunotherapy focuses on both primary and metastatic tumors. According to our results, the three combinations here could efficiently inhibit distant tumors and prevent lung metastasis. It was observed that the formation of the T_EM_ cell frequency was strongly induced in mice treated with CS@P plus laser irradiation and aPD-L1.

## Conclusions

In summary, our study herein demonstrated that the CCF-mediated PTT, NR2F6 gene silencing, and aPD-L1 combination treatment generated an attractive concept in HCC therapy that could stimulate effective synergistic therapeutic immunotherapy to inhibit tumor growth. Our pH-sensitive CCF nanoparticles are responsive to the slightly acidic tumor extracellular environment to improve tumor accumulation and cellular uptake efficiency and effectively deliver *Nr2f6* siRNA into H22 tumor cells and T cells to degrade mRNA. Additionally, mild CCF-mediated PPT can efficiently remodel the immunosuppressive microenvironment of HCC by inducing ICD and TIL infiltration, which amplifies aPD-L1 immunotherapy and genetic NR2F6 ablation and further prevents CTLs from adopting a dysfunctional/exhausted phenotype. The results showed that this siRNA-assisted assembly strategy together with PTT and aPD-L1 can significantly suppress the growth of irradiated primary tumors and non-irradiated distant tumors. Furthermore, immunological responses stimulated by local treatment can enable the establishment of long-term immunological memory throughout the body, and thereby inhibit tumor metastasis. This NR2F6 gene ablation/PTT-mediated aPD-L1 immune augmentation approach can provide inspiration to extend the benefits of immunotherapy to more HCC patients.

## Supplementary Information

**Additional file 1: Figure S1.** Size distribution of CCF-LDH in three mediums. **Figure S2.** Stability tests of CCF-LDHs in PBS, water and 1640 by monitoring particle size for 7 days. Error bars stand for ± SD (n = 3). **Figure S3.** XRD pattern of CCF-LDHs nanosheets. **Figure S4.** EDS of the CCF-LDHs. **Figure S5.** ^1^H NMR spectrum of PEG-DMMA. **Figure S6.** FTIR spectra. **Figure S7.** HRTEM image of C@P (Scale bar = 100 nm). **Figure S8.** Temperature elevation of C@P (100 μg/mL) under 808 nm NIR laser with different irradiation power in pH 6.5. **Figure S9.** Thermal infrared images of C@P (100 μg/mL) in tubes. **Figure S10.** HRTEM image of CCF-LDHs-siRNA. Scale bar = 100 nm. **Figure S11.** Zeta potential of CCF-LDHs-siRNA. **Figure S12.** Stability tests of CS@P in PBS, water and 1640 (with and without FBS) by monitoring particle size for 7 days. Digital photos of CCF-LDHs, C@P, and CS@P in 1640 with 10% FBS (bottom left). Error bars stand for ± SD (n = 3). **Figure S13.** The confocal images of intracellular localization of fluorescent FITC-CS@P in H22 and T cells for 4 h. Scale bar = 10 μm. **Figure S14.** The fluorescence intensity of FITC in H22 cell after the indicated treatments. **Figure S15.** In vitro cell viability of H22 tumor cells incubated with C@P for 24h. **Figure S16.** In vitro cell viability of H22 tumor cells incubated with C@P for 24h with laser irradiation (300s, 1 W cm^−2^). **Figure S17.** Fluorescence images of H22 tumor-bearing mice pre, 6, 12, 24, and 48 postinjection with CS or CS@P. **Figure S18.** Ex vivo fluorescence images of harvested organs and tumors at 48 h after the indicated treatments. **Figure S19.** In vivo MR imaging before and after intravenous injection of CS@P (dose:1 mg kg^−1^) within 48h. **Figure S20.** Thermal images of tumor-bearing mice injected with PBS and C@P, respectively, with irradiation. **Figure S21.** The tumor digital images obtained from H22 tumor-bearing mice on day 21 after treatment treated with PBS, aPD-L1, C@P, C@P + Laser, C@P + Laser + aPD-L1, CS@P, CS@P + Laser, and CS@P + Laser + aPD-L1. **Figure S22.** Survival curves of H22 tumor-bearing mice in different groups (n = 6). **Figure S23.** Change in body weight after the indicated treatment. **Figure S24.** H&E staining of the major organs. **Figure S25.** Flow cytometry analysis of the CD44 and CD62L expressions on primary tumor sites (a) and spleens (b) with various treatments (gated on CD3^+^CD8^+^).

## Data Availability

All data generated or analyzed during this study are included in this published article and its additional file.

## Abbreviations

HCC: Hepatocellular carcinoma
ICB: Immune checkpoint blockade
TME: Tumor microenvironment
PTT: Photothermal therapy
ICD: Immunogenic cell death
DCs: Dendritic cells
TILs: Tumor-infiltrating T lymphocytes
NR2F6: Nuclear receptor subfamily 2, group F, member 6
IL-2: Interleukin-2
IFN-γ: Interferon-γ
TNF-α: Tumor necrosis factor-α
aPD-L1: Anti-PD-L1
LDHs: Layered double hydroxide nanoparticles
PEG: Polyethylene glycol
DMMA: Dimethylmaleic anhydride
CCF-LDHs: CoCuFe-LDHs
CS@P: PEG-DMMA-coated CCF-LDHs loaded with NR2F6-siRNA
HTLs: Helper T lymphocytes
CTLs: Cytotoxic T lymphocytes
MRI: Magnetic resonance imaging
T_CM_ cells: Central memory T cells
T_EM_ cells: Effector memory T cells
FBS: Fetal bovine serum
