# Supplementary material for: Ultra-thin layered double hydroxide-mediated photothermal therapy combine with asynchronous blockade of PD-L1 and NR2F6 inhibit hepatocellular carcinoma
Source: J Nanobiotechnology. 2022 Jul 30;20:351. doi: 10.1186/s12951-022-01565-9 (PMC9338598; doi:10.1186/s12951-022-01565-9)
Supplement: Supplementary file 1 — Additional file 1: Figure S1. Size distribution of CCF-LDH in three mediums. Figure S2. Stability tests of CCF-LDHs in PBS, water and 1640 by monitoring particle size for 7 days. Error bars stand for ± SD (n = 3). Figure S3. XRD pattern of CCF-LDHs nanosheets. Figure S4. EDS of the CCF-LDHs. Figure S5. 1H NMR spectrum of PEG-DMMA. Figure S6. FTIR spectra. Figure S7. HRTEM image of C@P (Scale bar = 100 nm). Figure S8. Temperature elevation of C@P (100 μg/mL) under 808 nm NIR laser with different irradiation power in pH 6.5. Figure S9. Thermal infrared images of C@P (100 μg/mL) in tubes. Figure S10. HRTEM image of CCF-LDHs-siRNA. Scale bar = 100 nm. Figure S11. Zeta potential of CCF-LDHs-siRNA. Figure S12. Stability tests of CS@P in PBS, water and 1640 (with and without FBS) by monitoring particle size for 7 days. Digital photos of CCF-LDHs, C@P, and CS@P in 1640 with 10% FBS (bottom left). Error bars stand for ± SD (n = 3). Figure S13. The confocal images of intracellular localization of fluorescent FITC-CS@P in H22 and T cells for 4 h. Scale bar = 10 μm. Figure S14. The fluorescence intensity of FITC in H22 cell after the indicated treatments. Figure S15. In vitro cell viability of H22 tumor cells incubated with C@P for 24h. Figure S16. In vitro cell viability of H22 tumor cells incubated with C@P for 24h with laser irradiation (300s, 1 W cm−2). Figure S17. Fluorescence images of H22 tumor-bearing mice pre, 6, 12, 24, and 48 postinjection with CS or CS@P. Figure S18. Ex vivo fluorescence images of harvested organs and tumors at 48 h after the indicated treatments. Figure S19. In vivo MR imaging before and after intravenous injection of CS@P (dose:1 mg kg−1) within 48h. Figure S20. Thermal images of tumor-bearing mice injected with PBS and C@P, respectively, with irradiation. Figure S21. The tumor digital images obtained from H22 tumor-bearing mice on day 21 after treatment treated with PBS, aPD-L1, C@P, C@P + Laser, C@P + Laser + aPD-L1, CS@P, CS@P + Laser, and CS@P [file 12951_2022_1565_MOESM1_ESM.docx]

**Ultra-thin Layered Double Hydroxide-Mediated Photothermal Therapy Combine with Asynchronous Blockade of PD-L1 and NR2F6 Inhibit Hepatocellular Carcinoma**

Figure S1. Size distribution of CCF-LDH in three mediums.

Figure S2. Stability tests of CCF-LDHs in PBS, water and 1640 by monitoring particle size for 7 days. Error bars stand for ± SD (n = 3).

Figure S3. XRD pattern of CCF-LDHs nanosheets.


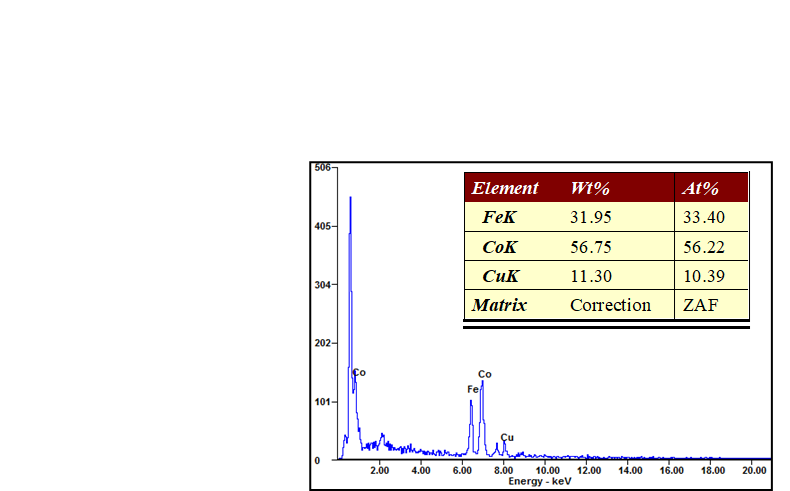


Figure S4. EDS of the CCF-LDHs.


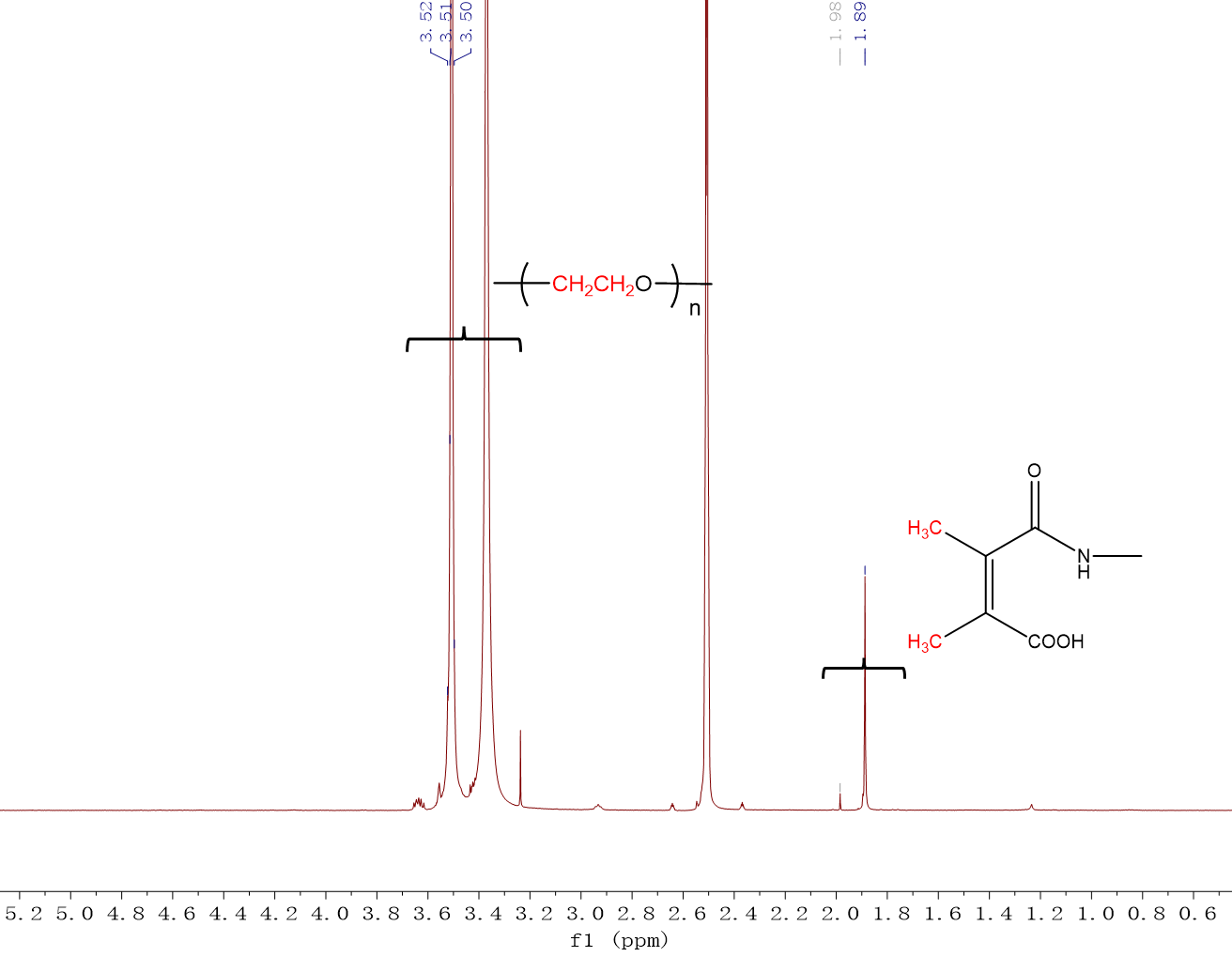


Figure S5. ^1^H NMR spectrum of PEG-DMMA.


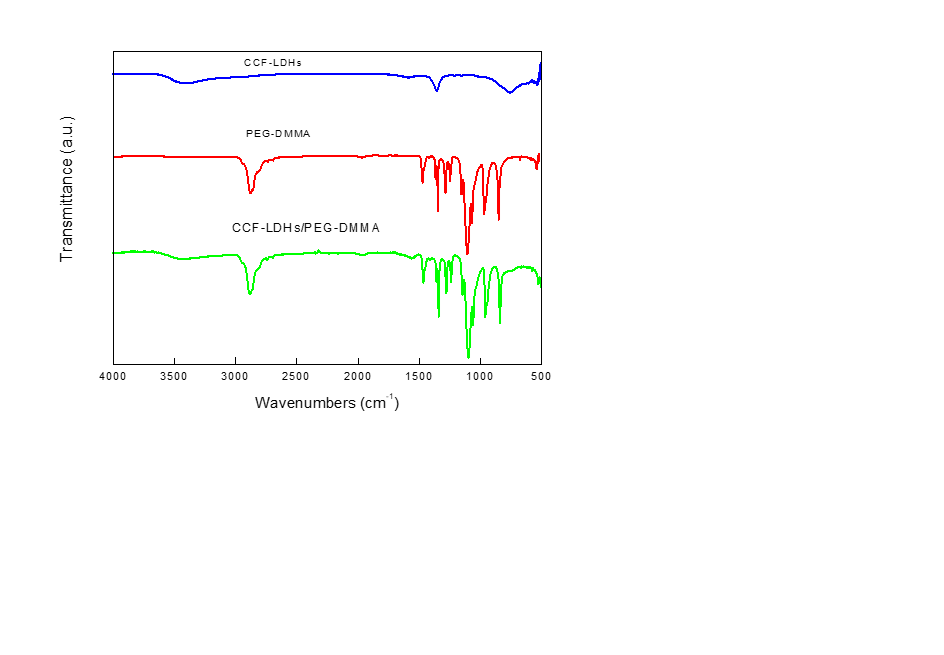


Figure S6. FTIR spectra.


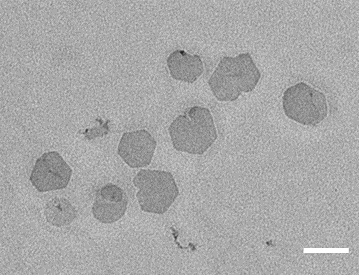


Figure S7. HRTEM image of C@P (Scale bar = 100 nm).

Figure S8. Temperature elevation of C@P (100 μg/mL) under 808 nm NIR laser with different irradiation power in pH 6.5.


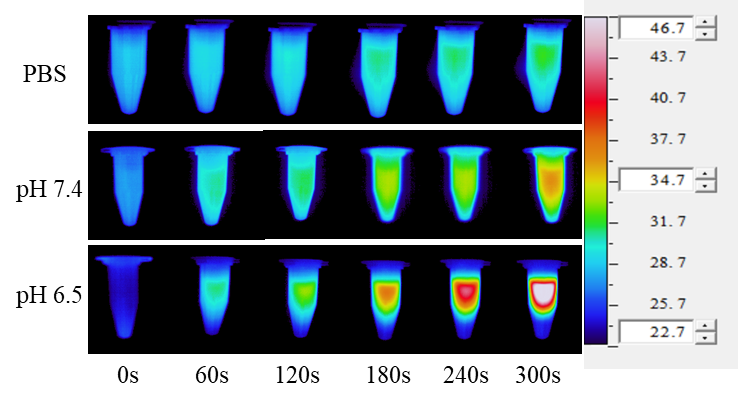


Figure S9. Thermal infrared images of C@P (100 μg/mL) in tubes.


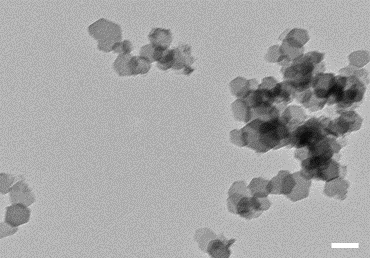


Figure S10. HRTEM image of CCF-LDHs-siRNA. Scale bar = 100 nm.

Figure S11. Zeta potential of CCF-LDHs-siRNA.


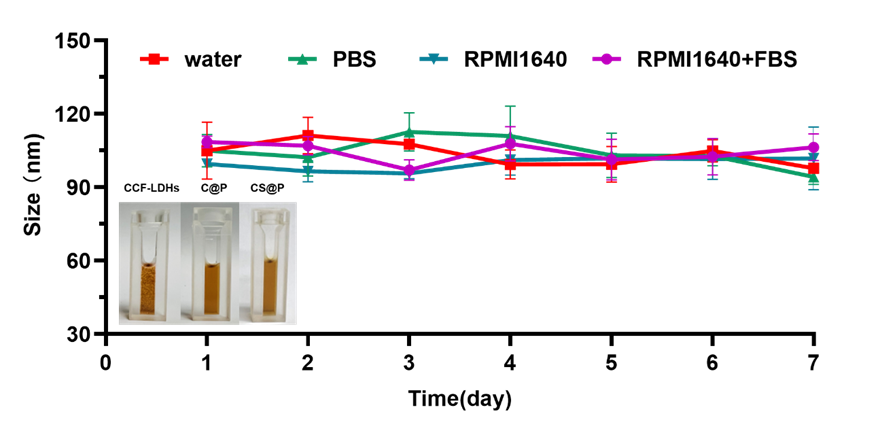


Figure S12. Stability tests of CS@P in PBS, water and 1640 (with and without FBS) by monitoring particle size for 7 days. Digital photos of CCF-LDHs, C@P, and CS@P in 1640 with 10% FBS (bottom left). Error bars stand for ± SD (n = 3).


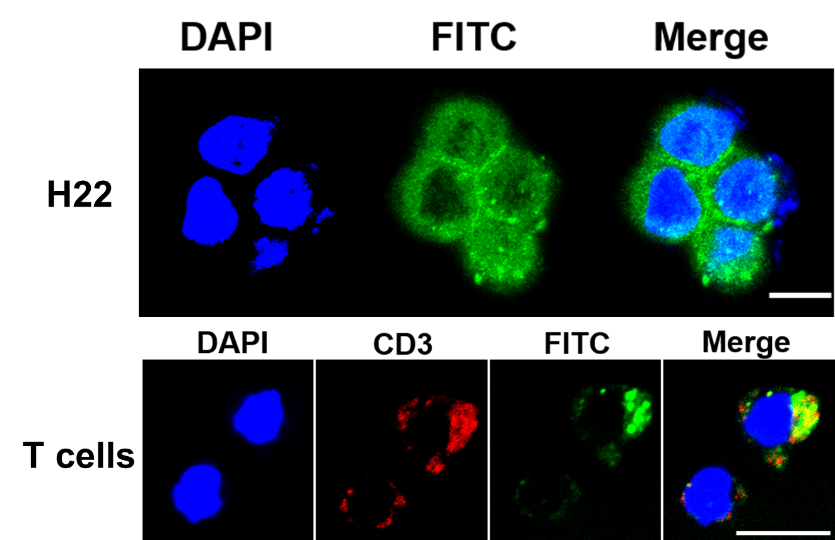


Figure S13. The confocal images of intracellular localization of fluorescent FITC-CS@P in H22 and T cells for 4 h. Scale bar = 10 μm.

**
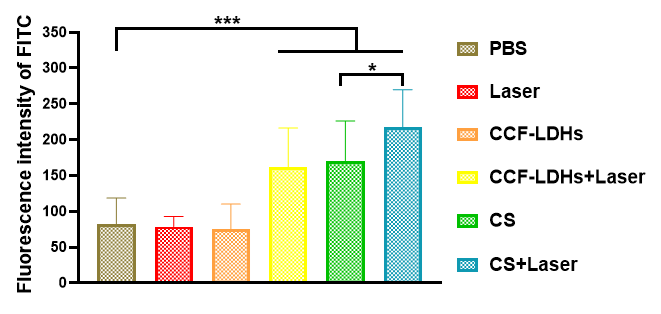
**

Figure S14. The fluorescence intensity of FITC in H22 cell after the indicated treatments.

Figure S15. In vitro cell viability of H22 tumor cells incubated with C@P for 24h.

Figure S16. In vitro cell viability of H22 tumor cells incubated with C@P for 24h with laser irradiation (300s, 1 W cm^−2^).


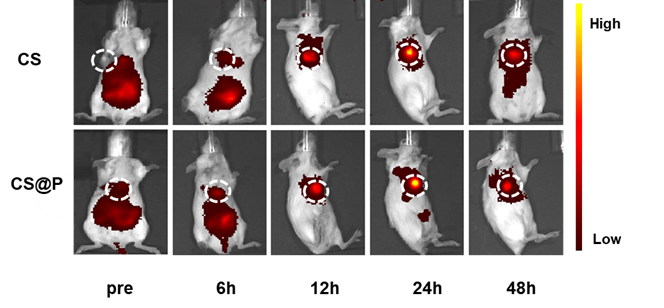


Figure S17. Fluorescence images of H22 tumor-bearing mice pre, 6, 12, 24, and 48 postinjection with CS or CS@P.


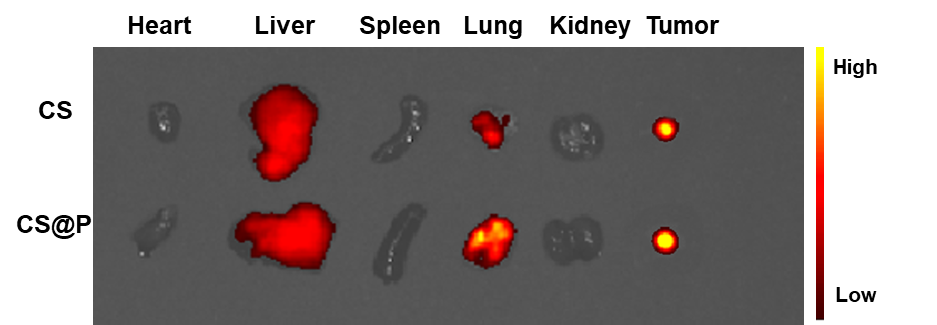


Figure S18. Ex vivo fluorescence images of harvested organs and tumors at 48 h after the indicated treatments.


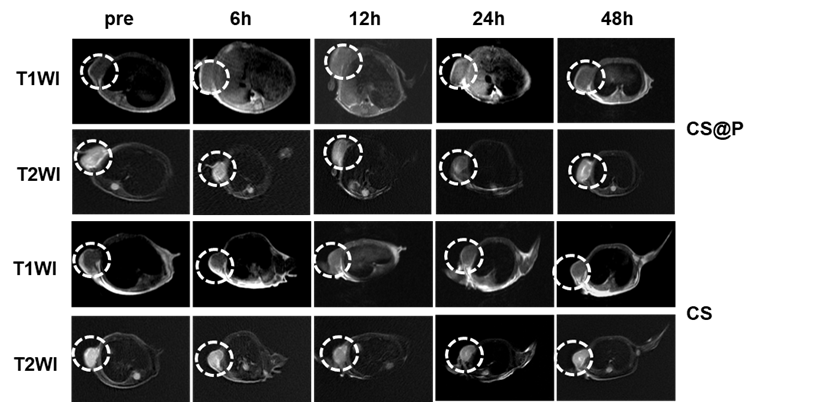


Figure S19. In vivo MR imaging before and after intravenous injection of CS@P (dose:1 mg kg^−1^) within 48h.


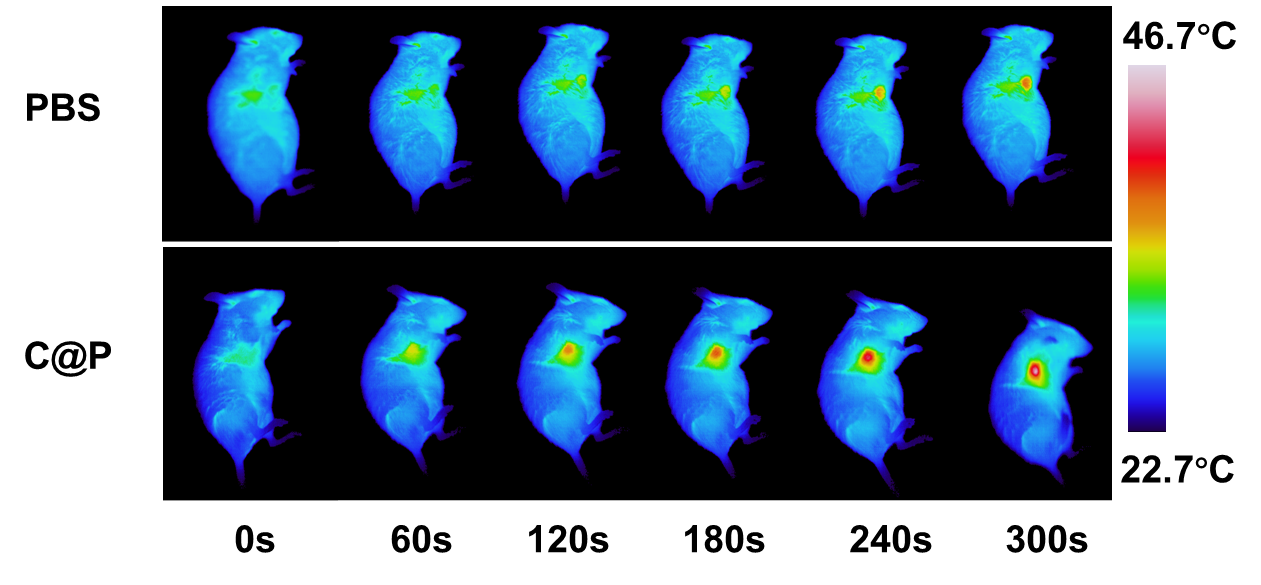


Figure S20. Thermal images of tumor-bearing mice injected with PBS and C@P, respectively, with irradiation.


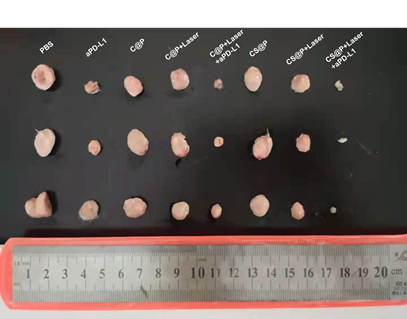


Figure S21. The tumor digital images obtained from H22 tumor-bearing mice on day 21 after treatment treated with PBS, aPD-L1, C@P, C@P + Laser, C@P + Laser + aPD-L1, CS@P, CS@P + Laser, and CS@P + Laser + aPD-L1.

 Figure S22. Survival curves of H22 tumor-bearing mice in different groups (n = 6).

Figure S23. Change in body weight after the indicated treatment.


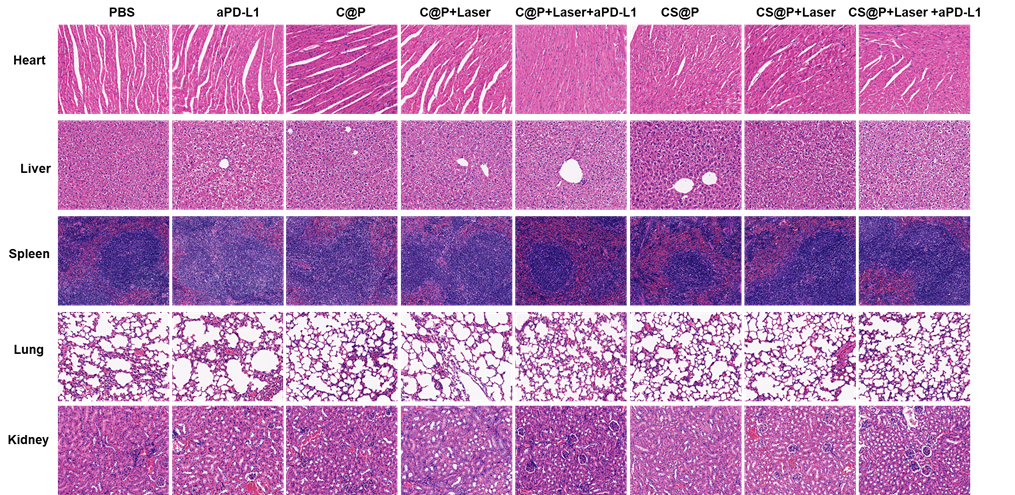


Figure S24. H&E staining of the major organs.


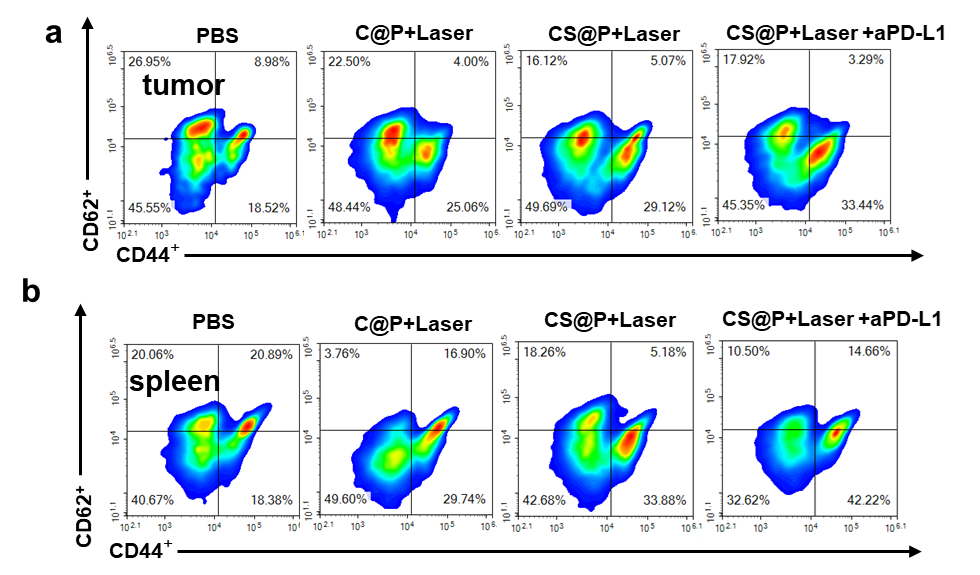


Figure S25. Flow cytometry analysis of the CD44 and CD62L expressions on primary tumor sites (a) and spleens (b) with various treatments (gated on CD3^+^CD8^+^).
